# Evolution of homo‐oligomerization of methionine S‐adenosyltransferases is replete with structure–function constrains

**DOI:** 10.1002/pro.4352

**Published:** 2022-06-16

**Authors:** Daniel Kleiner, Ziva Shapiro Tuchman, Fannia Shmulevich, Anat Shahar, Raz Zarivach, Mickey Kosloff, Shimon Bershtein

**Affiliations:** ^1^ Department of Life Sciences Ben‐Gurion University of the Negev Beer‐Sheva Israel; ^2^ The Department of Human Biology, Faculty of Natural Sciences University of Haifa Haifa Israel; ^3^ Ilse Katz Institute for Nanoscale Science & Technology Ben‐Gurion University of the Negev Beer‐Sheva Israel; ^4^ Macromolecular Crystallography and Cryo‐EM Research Center, The National Institute for Biotechnology in the Negev Ben‐Gurion University of the Negev Beer‐Sheva Israel

**Keywords:** dihedral homotetramer, methionine S‐adenosyltransferase, protein interface, quaternary structure evolution, stopped‐flow kinetics, structure‐based energy calculation, X‐ray crystallography

## Abstract

Homomers are prevalent in bacterial proteomes, particularly among core metabolic enzymes. Homomerization is often key to function and regulation, and interfaces that facilitate the formation of homomeric enzymes are subject to intense evolutionary change. However, our understanding of the molecular mechanisms that drive evolutionary *variation* in homomeric complexes is still lacking. How is the diversification of protein interfaces linked to variation in functional regulation and structural integrity of homomeric complexes? To address this question, we studied quaternary structure evolution of bacterial methionine S‐adenosyltransferases (MATs)—dihedral homotetramers formed along a large and conserved dimeric interface harboring two active sites, and a small, recently evolved, interdimeric interface. Here, we show that diversity in the physicochemical properties of small interfaces is directly linked to variability in the kinetic stability of MAT quaternary complexes and in modes of their functional regulation. Specifically, hydrophobic interactions within the small interface of *Escherichia coli* MAT render the functional homotetramer kinetically stable yet impose severe aggregation constraints on complex assembly. These constraints are alleviated by electrostatic interactions that accelerate dimer‐dimer assembly. In contrast, *Neisseria gonorrhoeae* MAT adopts a nonfunctional dimeric state due to the low hydrophobicity of its small interface and the high flexibility of its active site loops, which perturbs small interface integrity. Remarkably, in the presence of methionine and ATP, *N. gonorrhoeae* MAT undergoes substrate‐induced assembly into a functional tetrameric state. We suggest that evolution acts on the interdimeric interfaces of MATs to tailor the regulation of their activity and stability to unique organismal needs.

## Abstract 2

PDB Code(s): 7R2W;7R3B;

## 1 INTRODUCTION

The astounding complexity of cellular processes found even in the most minimalistic of organisms is rooted in the capacity of protein molecules to assemble into higher‐order functional oligomers.^1^ Oligomeric proteins include homomers, comprised of self‐interacting copies of a single subunit, and heteromers, composed of distinct polypeptide chains. Analysis of the statistical properties of the interacting protein‐like surfaces led to a conclusion that homomers are favored over heteromers because self‐complementary homodimeric interfaces are energetically more favorable than the heterodimeric ones.^2^, ^3^ Homomers indeed constitute a significant proportion of protein complexes in the cell, particularly in single‐cell organisms, where at least 50% of protein complexes appear to be homomeric.^1^, ^4^, ^5^ Homomers are involved in all major cellular processes, including gene expression, metabolism, transport, and signal transduction.^6^ Importantly, homomerization can generate new functionalities by forming catalytic and ligand‐binding sites directly at homomeric interfaces,^7^, ^8^, ^9^, ^10^, ^11^, ^12^ or regulate function via a concentration‐dependent transition between discrete oligomeric states.^13^, ^14^ Homomers are known to undergo reversible transitions between discrete conformations that preserve the oligomeric state and account for cooperative binding and allosteric mechanisms.^8^, ^11^

Given the prevalence of homomers in nature and their often intimate relationship with physiological functions, it is not surprising that numerous studies have been conducted over the years to understand the molecular determinants and evolutionary mechanisms of homomerization.

In‐depth analyses of selected protein interfaces have revealed that size, shape, and physicochemical complementarity are key determinants that drive the formation of homomeric complexes.^15^, ^16^, ^17^, ^18^, ^19^, ^20^, ^21^ Importantly, hydrophobic and electrostatic interactions, the major forces that stabilize intersubunit interactions within homomers and control the rate of complex (dis)assembly,^22^, ^23^, ^24^, ^25^, ^26^, ^27^, ^28^ have also been implicated in protein aggregation.^29^, ^30^, ^31^, ^32^, ^33^ This suggests that homomeric interface evolution imposes constraints on folding and assembly of individual subunits. Comparisons across multiple and highly distinct protein families revealed that homomeric interfaces can differ, with no clear correlation between interface size and binding free energy.^34^ However, within the context of closely related homologous homomers, this correlation was substantial, with more evolutionary‐ancient interfaces being larger and having stronger interactions than more recently evolved interfaces.^35^ Further, it was shown that the hierarchy in interface sizes and binding strength limits homomer assembly to a single dominant path. Specifically, assembly proceeds via energetically favorable intermediate subcomplexes that mimic the evolutionary precursors of homomer formation.^36^, ^37^

**FIGURE 1:**
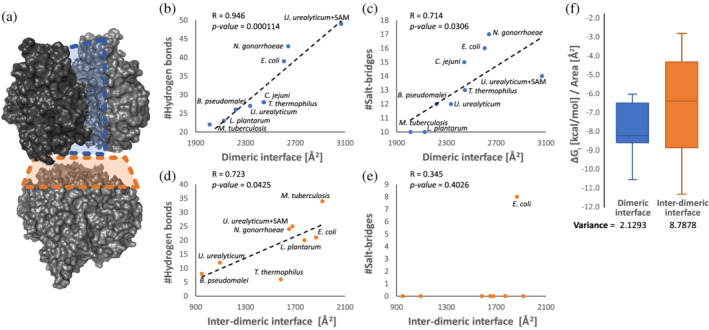
Comparison of the dimeric and interdimeric methionine S‐adenosyltransferases (MAT) interfaces reveals distinct modes of selection. (a) *Escherichia coli* MAT homotetramer (PDB ID 1P7L). The individual subunits are colored in shades of gray. The large (dimeric interface) is shown in blue. The small (interdimeric interface) is shown in orange. The number of hydrogen bonds (b) and salt‐bridges (c) in the dimeric interfaces correlates linearly with the interface size. (d) Correlation between the number of H‐bonds and small interface sizes is less significant. (e) Lack of correlation between the number of salt‐bridges and small interface sizes. (f) Distributions of the large and small interface hydrophobicities calculated as a ratio between free energy change in solvation energy (Δ*G*_*i*_, cal/mol) and interface size (Å^2^). R‐Pearson linear correlation coefficient; *p*‐values of the Pearson's correlations are calculated with analysis of variance

Despite the important evolutionary and mechanistic insights generated by these studies, we still lack a basic understanding of the molecular mechanisms that drive the evolutionary *variation* in homomeric complexes, even among closely related homologues. Specifically, it is not known how divergence in the physicochemical determinants of interfaces is linked to variation in functional regulation and to the structural integrity of homomeric quaternary structures. Moreover, it is not clear how the constraints imposed by interface evolution on folding and assembly of individual protomers influence the diversification of homomers. Here we address these questions by analyzing the quaternary structures of bacterial methionine S‐adenosyltransferases (MATs). MATs are an essential and ubiquitous enzyme family found in all domains of life.^38^ MATs catalyze the condensation of ATP with methionine to generate S‐adenosylmethionine (SAM)—an essential molecule involved in numerous biological processes, including RNA, DNA, protein and small molecule methylation, polyamine synthesis, and production of enzyme cofactors.^39^ Analysis of available crystal structures reveals that bacterial MATs are predominantly dihedral homotetramers (D_2_ symmetry group), that is, comprised of dimers of dimers^40^ (Figure 1a). MAT monomers pair via a large and flat hydrophobic interface. Two deep cavities harboring active sites are located directly in this dimeric interface, making the homodimer the obligatory functional unit. Homodimers, in turn, pair via a smaller interface to form a homotetramer (Figure 1a). The emergence of MAT dimers was likely an earlier adaptive event as it led to formation of two active sites within the large interface. However, the significance of the more recent evolutionary association of MAT dimers into a tetramer is less clear, particularly in light of the facts that (i) dimeric MATs have also been reported^41^, ^42^; and (ii) the assigned biological assemblies of approximately half of the available tetrameric MAT structures from bacteria in the Protein Data Bank (PDB) are dimers (Table 1). We found that while the large (interdimeric) interface of bacterial MATs is subject to tight control by purifying selection, the small (dimeric) interface constitutes a playground for intense evolutionary diversification. Using X‐ray crystallography, stopped‐flow kinetics, structure‐based per‐residues energy calculations, and other analytical approaches we identified physicochemical determinants within small interfaces that are responsible for unique structural and functional properties of MAT complexes. Specifically, we show that high hydrophobicity of the small interface leads to kinetic stability of *Escherichia coli* MAT homotetramers, but also incurs severe aggregation constraints on dimer‐to‐tetramer assembly, and, possibly, on the folding of individual protomers. We also demonstrate that the low hydrophobicity of the small interface and the high flexibility of the active site loops sustain *Neisseria gonorrhoeae* MAT in a dimeric state in the absence of substrates but trigger functional tetramerization upon addition of ATP and methionine. Our data suggest that evolution acts on the interdimeric interfaces of MATs to tailor their activity and structural integrity to the unique metabolic needs of bacteria, while preserving the integrity of the functionally crucial large interface.

**TABLE 1:** Size and composition of bacterial methionine S‐adenosyltransferases (MAT) interfaces calculated by protein interfaces, surfaces, and assemblies

Strain (PDB ID)	Class	Biological assembly^a^	% Sequence identity to *E. coli*	Interface	Area (Å^2^)	ΔGi^b^ (kcal/mol)	Δ*G*_i_ ^c^ /area	#Res^d^	#HB^e^	#SB^f^
*E. coli* (1P7L)	Gammaproteobacteria	Tetramer	100%	Large	2,608	−22.5	−8.6	86	39	16
				Small	1867	−16.9	−9.1	62	21	8
*Neisseria gonorrhoeae* (5T8S)	Betaproteobacteria	Dimer	68%	Large	2,643	−22.5	−8.5	80	43	17
				Small	1,651	−4.7	−2.8	59	24	0
*Burkholderia pseudomallei* (3IML)	Betaproteobacteria	Dimer	70%	Large	2,221	−23.5	−10.6	71	26	12
				Small	949	−8.4	−8.9	42	8	0
*Campylobacter jejuni* (4LE5)^g^	Campylobacteria	Dimer	41%	Large	2,449	−21.3	−8.7	79	28	13
				Small						
*Mycobacterium tuberculosis* (3TDE)	Actinomycetia	Tetramer	59%	Large	2012	−12.2	−6.1	70	22	10
				Small	1918	−13.8	−7.2	70	34	0
*Lactiplantibacillus plantarum* (7R3B)^h^	Bacilli	Dimer	62%	Large	2,126	−13.9	−6.5	72	23	10
				Small	1773	−10	−5.6	59	20	0
*Thermus thermophilus* (5H9U)	Deinococci	Tetramer	60%	Large	2,444	−17.1	−7.0	81	28	15
				Small	1,584	−18	−11.4	64	6	0
U. Urealyticum (6RJS)	Mollicutes	Dimer	44%	Large	2,337	−19.3	−8.3	72	27	12
				Small	1,094	−4.9	−4.5	45	12	0
*Ureaplasma Urealyticum* + S‐adenosylmethionine (6RKC)	Mollicutes	Tetramer	44%	Large	3,069	−19.8	−6.5	94	49	14
				Small	1,677	−6.7	−4.0	51	25	0
*E. coli* E67K K98Q (7R2W)^h^	Gammaproteobacteria	Tetramer	99.5%	Large	2,245	−16.4	−7.3	72	34	12
				Small	2055	−24.2	−11.8	74	10	4

^a^ Biological assembly assigned by the authors in the PDB.

^b^ Δ*G*_*i*_ is a free energy change in solvation energy. It corresponds to hydrophobic interactions within the interfaces and does not include the contribution of hydrogen bonds and salt bridges across the interface.

^c^ Contribution of hydrophobicity to the free energy of interaction per 1 Å^2^ of interface is calculated as a ratio between the free energy change in solvation energy (Δ*G*_*i*_, cal/mol) and interface size (Å^2^).

^d^ Number of residues.

^e^ Number of hydrogen bonds.

^f^ Number of salt‐bridges.

^g^ *C. jejuni* MAT does not form a tetramer in the crystal structure.

^h^ X‐ray crystal structures solved in this work.

## 2 RESULTS

### 2.1 The dimeric and interdimeric interfaces of bacterial MATs are subject to different modes of evolution

**FIGURE 2:**
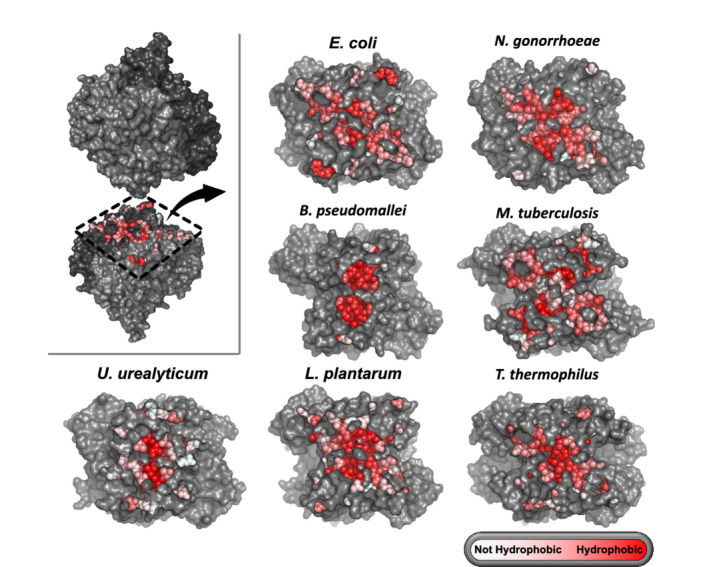
Surface representation of the small (interdimeric) interfaces of bacterial methionine S‐adenosyltransferases. Residues directly forming the interaction are colored according to their hydrophobicity^44^

To better understand the structural diversity of bacterial MATs, we used the Protein Interfaces, Surfaces, and Assemblies (PISA)^43^ server to analyze the interface size, composition, and types of interactions within bacterial MATs with known structures (Table 1, Figure 1b–f). MAT structures from only a few bacterial phyla are currently publicly available, including Proteobacteria, Actinobacteria, Deinococcota, and Mycoplasmatota (Table S1). To enrich the structural dataset, we determined the crystal structure of MAT from *Lactiplantibacillus planatrum* at 2.82 Å resolution, making it the only current MAT representative of the Bacillota phylum (Table S2, Figure S1), and added this structure to analysis (Table 1). We found that the surface area of the dimeric (large) interfaces scales up significantly with the number of H‐bonds (*p*‐value = .0001) and salt‐bridges (*p*‐value = .031)—a known feature of homodimeric interfaces^16^ (Figure 1b,c and Table 1). Importantly, the correlations between the surface area of the dimeric (large) interfaces and the number of H‐bonds (*p*‐value of the Pearson's correlation = .0001, analysis of variance [ANOVA] test) and salt‐bridges hold despite the distant phylogenetic and ecological relationships between the bacteria from which the MATs originate, including disease‐causing parasitizing bacteria (*N. gonorrhoeae* and *Ureaplasma urealyticum*), and the hyperthermophilic bacterium *Thermus thermophilus*. In contrast, the correlation between the number of H‐bonds and the size of the inter‐dimeric (small) interfaces is less significant (*p*‐value of the Pearson's correlation = .043, ANOVA test, Figure 1d), and no correlation can be found between the number of salt‐bridges and the interface size; we note the conspicuous absence of salt bridges in the inter‐dimeric interfaces of all known bacterial MATs, with the exception of *E. coli* MAT (Figure 1e and Table 1). Next, we compared the distribution of hydrophobicity of the dimeric and interdimeric MAT interfaces, calculated as a ratio between the free energy change in solvation energy (a proxy to contribution of hydrophobic interaction to the interaction energy) and interface size, Δ*G*_*i*_/area (Figure 1f and Table 1). We found that the distribution of solvation energy per 1 Å^2^ (in units of cal/mol/Å^2^) of the dimeric interfaces appears rather narrow (mean − 7.9 cal/mol/Å^2^; variance = 2.1). Conversely, the variance in Δ*G*_*i*_/area among interdimeric interfaces is large (mean − 6.7 cal/mol/Å^2^; variance = 8.8), with over fourfold difference between MATs from *N. gonorrhoeae* and *T. thermophilus* (Table 1). Finally, we compared the variability in geometry of large and small MAT interfaces across the available structures. While large MAT interfaces clearly share a similar geometry and hydrophobicity pattern (Figure S2), these features within small MAT interfaces are strikingly distinct (Figure 2). Collectively, these analyses suggest that while the homo‐dimerization of MAT is subject to a tight control of purifying selection, the homo‐tetramerization via interdimeric interfaces is under intense evolutionary diversification. It is plausible that at least some of the diversity observed in the MAT inter‐dimeric interfaces is a result of adaptive evolution that adjusts the control over catalytic activity and structural integrity of the homomeric structure to the physiological/environmental needs of bacteria.

### 2.2 Variability in MAT interdimeric interfaces is linked to differences in the kinetic stability of the quaternary structures

**FIGURE 3:**
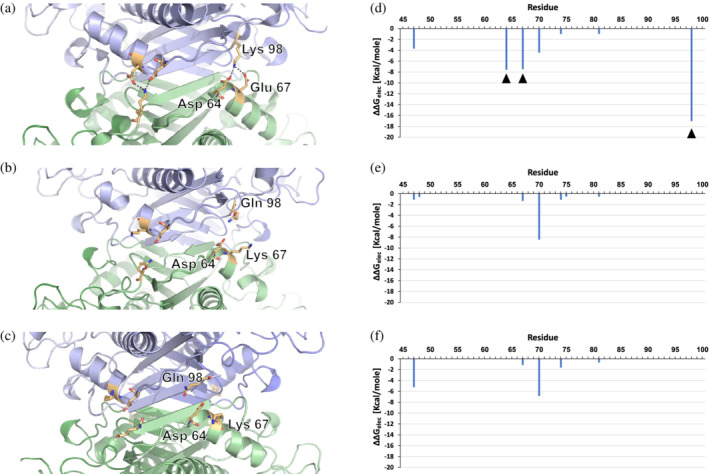
Per‐residue energy contributions of the electrostatic interactions within small interface. Cartoon close‐up of the small (interdimeric) interface in EcMAT (PDB ID 1P7L) (a), NgMAT (PDB ID 5T8S) (b), and EcMATmut (PDB ID 7R2W) (c) Each dimer is colored in blue and green. Salt‐bridge‐forming residues in EcMAT and residues occupying homologous positions in NgMAT and EcMATmut are shown in sticks (nitrogen atoms are shown in blue and oxygen atoms in red). (d–f) Structure‐based per‐residue calculation of the electrostatic energy contributions of electrostatic interactions (ΔΔ*G*_elec_) from the side‐chains of residues interacting across the small interface of EcMAT (d), NgMAT (e), and EcMATmut (f) (see Section 4 and Figure S5). Black triangles in (D) indicate the location of the salt‐bridge‐forming residues in EcMAT. Residues are numbered according to EcMAT

**FIGURE 4:**
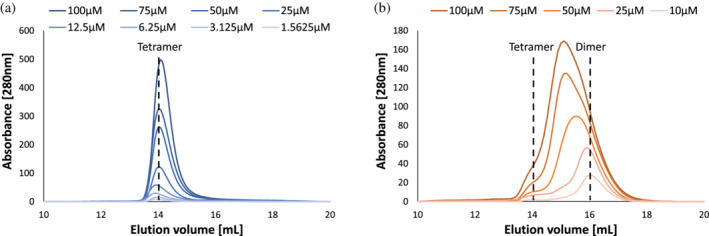
Size exclusion chromatography (SEC) analysis of methionine S‐adenosyltransferases (MAT) homomeric states in solution at a range of protein concentrations. (a) EcMAT (1.56–100 μM). (b) NgMAT (10–100 μM). The approximate molecular weight of the observed homomeric states was determined using titration of molecular standards (Figure S6)

**FIGURE 5:**
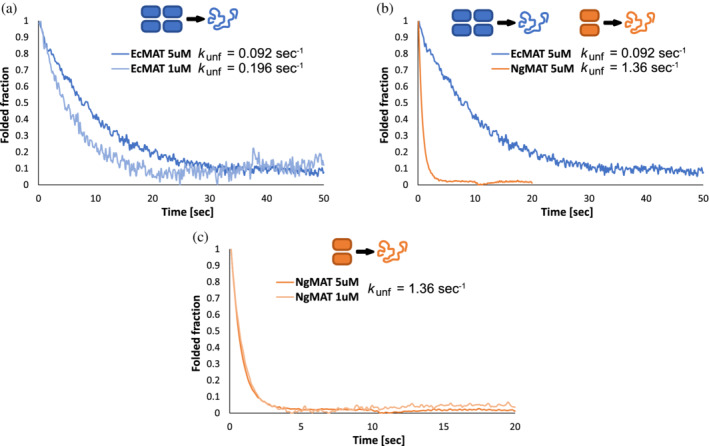
Stopped‐flow measurements of the urea‐induced apparent dissociation/unfolding rate (*k*_unf_) of methionine S‐adenosyltransferases (MAT) proteins. (a). Kinetics of EcMAT dissociation/unfolding at 1 μM (light blue) and 5 μM (dark blue) protein. (b). Comparison of the kinetics of dissociation/unfolding of EcMAT (blue trace) and NgMAT (orange trace) at 5 μM protein. (c) Kinetics of NgMAT dissociation/unfolding at 1 μM (light orange) and 5 μM (dark orange) protein. The homomeric native state is shown in rectangles above the figures. *k*_unf_ was derived by fitting the obtained traces to a single exponential (see Section 4)

How is the observed diversity in the molecular properties of small MAT interfaces related to the stability and assembly/disassembly dynamics of the tetrameric complex? To address this question, we focused on MATs from *E. coli* and *N. gonorrhoeae* (EcMAT and NgMAT, respectively). These proteins share 68.5% amino acid sequence identity, form homotetrameric complexes in all available crystal structures (Tables 1 and S1), and have structurally similar monomeric and dimeric structures—the root mean square deviation between the α‐carbon atoms of the monomers and dimers is 1.23 and 1.48 Å, respectively (Figure S3). The monomers from both proteins also exhibit a virtually identical thermodynamic stability, as determined by the urea‐induced equilibrium unfolding measurements performed with far‐UV circular dichroism (see Section 4 and Figure S4A‐C). However, close analysis of the physicochemical properties of the small (interdimeric) interfaces reveals a dramatic difference in the hydrophobic and electrostatic interactions between EcMAT and NgMAT (Table 1). Specifically, the contribution of hydrophobic interactions to the binding free energy per unit area (Δ*G*_i_/area) in the EcMAT small interface is threefold higher than that in NgMAT's (Table 1). Further, whereas the small interface of NgMAT has no salt‐bridges, the small interface of EcMAT contains eight: Lys98 in each EcMAT subunit forms a salt‐bridge network with Asp64 and Glu67 of the opposing subunit (Figure 3a). The corresponding homologous positions in NgMAT are occupied with Gln98, Asp64, and Lys67, thus precluding the formation of salt‐bridges (Figure 3b). Using structure‐based per‐residue energy calculations^45^ (see Section 4 and Figure S5A,B), we estimated that the net electrostatic contribution (ΔΔ*G*_elec_) of the identified salt‐bridges in the EcMAT small interface is exceedingly large and amounts to approximately −18 kcal/mol from Lys98, and approximately −7 kcal/mol from both Asp64 and Glu67 (Figures 3c and S5C). No such contribution can be found in the small interface of NgMAT (Figure 3d and S5C). We determined that such a dramatic difference in the physicochemical properties of the small interfaces is also manifested in difference between the homo‐oligomeric states of EcMAT and NgMAT in solution. Size exclusion chromatography (SEC) analysis of the purified proteins showed that when EcMAT is diluted from a concentrated stock (300 μM), pre‐incubated in a diluted state for an hour and then injected into a SEC column, it preserves its homotetrameric state at a concentration as low as 1.6 μM (see Section 4, Figures 4a and S6). In contrast, under identical experimental conditions, NgMAT assumes a fully dimeric state already at 10 μM. NgMAT persists in a predominantly dimeric state at a concentration as high as 100 uM (Figure 4b). Given the high hydrophobicity of the interdimeric interface of EcMAT, we hypothesized that persistence of the tetrameric state of EcMAT upon dilution is the result of slow tetramer‐dimer dissociation rate constant (*k*_off[T→D]_), or, in other words, a high kinetic stability of the EcMAT homotetrameric structure. To validate this conjecture, we measured the apparent rate of EcMAT and NgMAT urea‐induced disassembly/unfolding by following a shift in Trp fluorescent signal using stopped‐flow (see Section 4). Far‐UV circular dichroism measurements demonstrated that upon exposure to 6 M urea, monomers of both EcMAT and NgMAT undergo an almost complete unfolding, as judged by the disappearance of secondary structure (Figure S4b,c). Thus, the amplitude of a change in the Trp fluorescence signal observed upon mixing MAT proteins with 6 M urea within a stropped‐flow instrument must also be a result of monomer unfolding. Yet, the apparent *rate* with which the fluorescent signal changes upon exposure to urea is controlled by the slowest process along the MAT disassembly/unfolding paths. Importantly, if complex disassembly is the rate limiting step, the measured apparent rates are expected to be sensitive to changes in protein concentrations (i.e., the higher the protein concentration the slower must be the obtained rate). Conversely, if monomer unfolding is the rate‐limiting step, the obtained apparent rates are expected to be concentration‐insensitive. We, therefore, compared the apparent rates of disassembly/unfolding (*k* ^app^_unf_) of EcMAT at two protein concentrations, 5 and 1 μM, and found a twofold rate enhancement at the lower protein concentration (*k* ^app^_unf_ = 0.196 s^−1^ at 1 μM EcMAT vs. *k* ^app^_unf_ = 0.096 s^−1^ at 5 μM) (Figure 5a). Given that EcMAT is predominantly tetrameric at 5 μM (Figure 4a), we conclude that the tetrameric state increases the kinetic stability of EcMAT, whereas the shift in the tetramer‐dimer equilibrium towards a dimeric state upon fivefold dilution reduces its kinetic stability. In line with this finding, the apparent rate of disassembly/unfolding of NgMAT at 5 μM is almost 15‐fold faster than that of EcMAT; 1.36 s^−1^ for NgMAT versus 0.092 s^−1^ for EcMAT at the same concentration (Figure 5b,c). Since NgMAT is fully dimeric already at 10 μM (Figure 4b), the difference in *k* ^app^_unf_ between EcMAT and NgMAT at 5 μM stems from the difference in the kinetic stabilities of a predominantly tetrameric EcMAT with a predominantly dimeric NgMAT. Moreover, a fivefold dilution of NgMAT, from 5 to 1 μM, did not change the apparent rate of unfolding, presumably because dimer‐monomer dissociation and monomer unfolding are coupled in this protein (Figure 5c). Collectively, these findings link the variability in the physicochemical properties of MAT interdimeric interfaces with the kinetic stability of the entire MAT quaternary structure.

### 2.3 Kinetic stabilization of MAT quaternary complex imposes constraints on folding and assembly of MAT protomers

**FIGURE 6:**
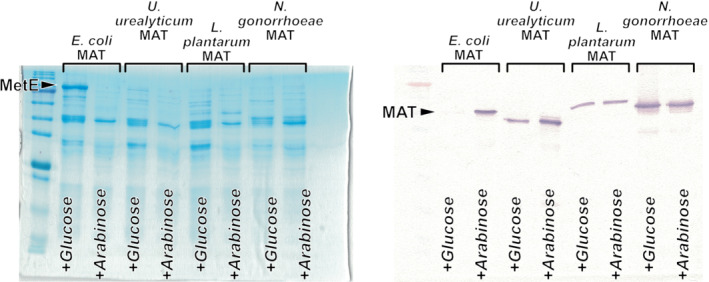
In vivo assay to assess methionine S‐adenosyltransferases (MAT) dependency on GroEL/ES. (a). Coomassie staining of the soluble proteome of MGM100 cells overexpressing MAT from (left‐to‐right) *Escherichia coli*, *Ureaplasma urealyticum*, *Lactiplantibacillus plantarum*, and *Neisseria gonorrhoeae* under the regime of reduced GroEL/ES levels (+glucose) and normal GroEL/ES levels (+arabinose). Note the high abundance of MetE, which is activated when intracellular SAM levels are low,^55^ in the case of *E. coli* MAT overexpression on a background of low GroEL/ES abundance (+glucose). (b). Western‐blot analysis of the proteome shown in (a) with custom‐raised anti‐MAT antibodies (see Section 4). Note the absence of soluble *E. coli* MAT on the background of low GroEL/ES abundance (+glucose)

Hydrophobic and electrostatic interactions have long been established as major factors that stabilize homomers and control the rate of complex (dis)assembly.^22^, ^23^, ^26^, ^27^, ^28^ However, these very physicochemical properties have also been implicated in driving protein aggregation^25^, ^29^, ^31^, ^33^, ^46^ and constituting molecular determinants recognized by chaperones.^47^ The contradictory positive correlation between the stability of protein interfaces and the aggregation propensity of protomers can increase the ruggedness of the folding landscape for protomers that form kinetically stable complexes and impose constraints on the kinetics of protomer folding and intermediate assembly. Given the substantial difference in the hydrophobic and electrostatic properties of the interdimeric interfaces in EcMAT and NgMAT, we reasoned that the evolutionary diversification of the homomeric states of these two proteins could have been shaped by distinct structural and kinetic constrains. To explore this possibility, we first turned to the folding requirements of the EcMAT and NgMAT protomers. Folding of EcMAT is known to be obligatory dependent on GroEL/ES chaperonins.^48^, ^49^ Since the chamber of GroEL is believed to accommodate proteins not larger than 60 kDa^50^ (but see also reports that demonstrate out‐of‐chamber GroEL folding assistance of much larger proteins^51^, ^52^), while the size of a single EcMAT subunit is approximately 42 kDA, it is probable that chaperonins assist in folding of only individual EcMAT subunits, but not higher homomeric states. Apart from EcMAT and MAT from *U. urealyticum*, an organism that has lost chaperonin encoding genes throughout genome reduction evolution,^53^ the folding dependence on MATs on chaperonins has not been established. We, therefore, set to determine whether the folding of NgMAT is chaperonin‐dependent. To this end, we used a previously established experimental system in *E. coli* to measure both in vivo solubility and activity of MAT proteins on a background of reduced GroEL/ES abundance.^48^, ^54^ We found that NgMAT is independent of chaperonins since it assumes a soluble and functional state in *E. coli*'s cytoplasm, regardless of GroEL/ES expression level (see Section 4 and Figure 6). We also found that MAT from *Lactiplantibacillus plantarum* (LpMAT) does not require chaperonins for folding and activity (Figure 6). Similarly to NgMAT, LpMAT assumes a predominantly dimeric state in solution (Figure S7), its interdimeric interface lacks salt‐bridges (Figure S5), and is less hydrophobic than EcMAT (Table 1). Although these findings do not allow us to state categorically that the distinct physicochemical properties of the interdimeric interfaces of EcMAT and NgMAT are directly responsible for the observed differential dependence of protomer folding on chaperonins, as GroEL/ES can recognize a different region in EcMAT other than a small interface, they do indicate that the evolutionary diversification of homo‐oligomeric states of EcMAT and NgMAT are governed by distinct constraints.

**FIGURE 7:**
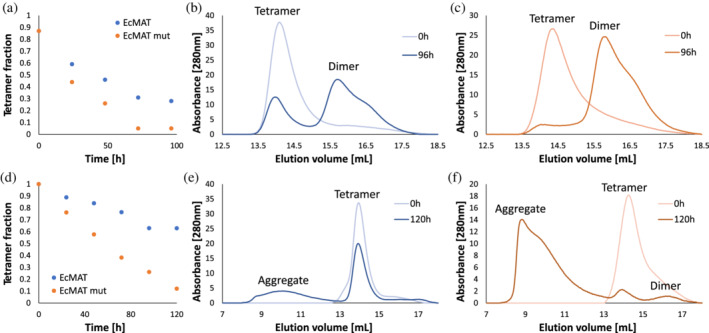
Size exclusion chromatography (SEC) analysis of methionine S‐adenosyltransferases (MAT) homomeric states upon prolonged incubation in the presence or absence of 50% glycerol. A,D. Reduction in the tetrameric fraction of EcMAT (blue) and EcMATmut (orange) along 96 hr of incubation in the presence of 50% glycerol (a), or along 120 hr of incubation in the absence of glycerol (d). (b,c) Distribution of the dimeric and tetrameric species of EcMAT (b) and EcMATmut (c) upon reaching equilibrium (96 hr in 50% glycerol). (e,f) Distribution of dimeric, tetrameric, and aggregated species of EcMAT (e) and EcMATmut (f) after 120 hr in absence of glycerol

Next, we attempted to determine the role of the salt‐bridges in the interdimeric interface of EcMAT in the assembly of the kinetically stable homotetrameric complex. Unlike hydrophobic interactions that operate at close proximity and preserve the integrity of the already formed interface, electrostatic interactions operate at longer ranges and are known to predominantly affect the association rate constant, *k*_on_.^26^, ^27^, ^28^ If the high hydrophobicity of the small EcMAT interface, whose role is to kinetically stabilize the dimer‐dimer interaction, also promotes aggregation of the dimeric units prior to their assembly into tetramers, charged residues in the interface can alleviate the aggregation constraint by enhancing the *k*_on_ of dimer–dimer interactions.^26^ To explore this hypothesis, we generated two reciprocal mutants, swapping the salt‐bridge‐forming residues in the EcMAT small interface (Lys98 and Glu67) with those of NgMAT (Gln98 and Lys67), and vice versa. Following purification of the *E. coli* mutant (EcMATmut) and the *N. gonorrhoeae* mutant proteins, we determined that the removal of the salt‐bridges did not affect the catalytic activity of EcMATmut (Figure S8), while the addition of salt‐bridge‐forming residues to NgMAT resulted in substantial aggregation (Figure S9). Additionally, we solved the crystal structure EcMATmut at 1.6 Å resolution (Table S2 and Figure 3c) and found that the protein assembles into a homotetramer that is highly structurally similar to EcMAT (Figure S10). PISA analysis of the small EcMATmut interface revealed that its hydrophobicity (Δ*G*_*i*_/area) increased by approximately 30% compared to that of EcMAT (11.8 vs. 9.0 cal/mol/Å^2^, respectively) (Table 1), whereas the net per‐residue electrostatic contribution (ΔΔ*G*_elec_) of EcMATmut was comparable to that of NgMAT (Figures 3f and S5c). Removal of the salt‐bridges did not change the kinetic stability of the tetrameric complex. This was anticipated because, as mentioned above, the electrostatic interactions contribute predominantly to the rate of complex formation (*k*_on_) rather than to the dissociation rate (*k*_off_) (Figure S11). To measure the impact of removing salt‐bridges on the rate of EcMAT dimer–dimer assembly, we diluted EcMAT and EcMATmut from concentrated stocks (~300 μM) to 10 μM and subjected the diluted samples to a prolonged incubation (100 hr) at 25°C. To prevent aggregation, the experiment was conducted in the presence of 50% glycerol (see Section 4). The protein samples were analyzed throughout the incubation period by size exclusion chromatography to determine whether equilibrium between the dimeric and tetrameric states has been reached. We found that immediately upon dilution, both EcMAT and EcMATmut protein samples contained predominantly tetrameric species, yet, with time, the dimeric species became more and more prominent in both proteins (Figure 7a). After approximately 75 hr, the equilibrium between dimers and tetramers was reached. Strikingly, while at equilibrium the tetrameric fraction of EcMAT constituted 30% (Figure 7b), the tetrameric fraction of EcMATmut was only 5% (Figure 7c). Based on these measurements we calculated the tetramer‐dimer equilibrium dissociation constant, *K*_d_ (see Section 4) and determined that the *K*_d_ of EcMAT was approximately 11‐fold lower than that of EcMATmut (8.2 vs. 90.3 μM, respectively). Under the assumption that both proteins have similar tetramer‐to‐dimer dissociation rate, *k*_off(T→D)_, as indeed suggested by the stopped‐flow kinetic measurements (Figure S11), we conclude that the over a magnitude difference in the *K*_d_ can be explained entirely by the contribution of the salt‐bridges in the EcMAT small interface to the rate of dimer‐dimer assembly, *k*_on(D‐D)_. Interestingly, removal of glycerol from the incubation buffer resulted in a dramatic and increasing aggregation of the dimeric fraction of EcMATmut, which, led to a loss of over 90% of the tetrameric fraction after 120 hr of incubation (Figure 7d,e). In contrast, the dimeric fraction of EcMAT was much less aggregation‐prone, with over 60% of the tetrameric fraction remaining under identical conditions (Figure 7f). These findings strongly support our hypothesis that the propensity for hydrophobic interactions within the EcMAT interdimeric interface renders the unassembled dimers aggregation‐prone. The presence of salt‐bridges in the interface enhances the rate of the homotetramerization and, thereby, reduces the risk of dimer aggregation.

**FIGURE 8:**
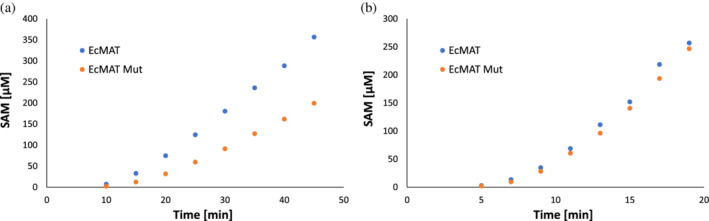
Rate of accumulation of SAM upon refolding of GuHCl‐unfolded methionine S‐adenosyltransferases (MAT) proteins in the presence of GroEL/ES. (a). Time dependent accumulation of S‐adenosylmethionine upon dilution of GuHCl‐unfolded EcMAT (blue) and EcMATmut (orange) proteins in a reaction mix containing substrates and chaperonins. (b). The same experiment as in (a) but with fivefold higher amounts of both proteins and chaperonins

Lastly, we explored how removal of the salt‐bridges from the EcMAT small interface affected the folding dependency of the protein on GroEL/ES. To this end, we followed the efficiency of refolding and assembly of the urea‐unfolded EcMAT and EcMATmut proteins in the presence of GroEL/ES in vitro (see Section 4). First, we determined that both proteins were fully dependent on chaperonins to attain a functional state from a denaturant‐unfolded state (Figure S12). Next, we compared the rate of accumulation of the reaction product, SAM, between EcMAT and EcMATmut upon 1:100 dilution of urea‐unfolded proteins into a reaction mix containing saturated amounts of methionine and ATP, an ATP‐regeneration system, and GroEL and GroES chaperonins at 1:2 and 1:4 M ratios, respectively, relatively to the EcMAT and EcMATmut protomer concentrations (see Section 4). We found that the rate of SAM accumulation by EcMATmut is approximately 60% lower than that of EcMAT (Figure 8a). Given that the catalytic activity of both proteins is identical (see Figure S8), the most probable explanations for the observed delay can be (i) a less‐efficient re‐folding of the mutant protomer by the chaperonins; and/or (ii) aggregation of dimeric EcMATmut due to inefficient assembly. To distinguish between these two possibilities, we repeated the experiment while increasing the concentration of the proteins fivefold. To maintain protomer:chaperonin ratios, we also increased the concentration of the chaperonins fivefold. If the delay is due to inefficient refolding of EcMATmut protomers, increasing the concentration of both proteins and chaperonins should have no effect. Conversely, if the delay is due to dimer aggregation, because of slow dimer‐dimer assembly, increasing protein concentrations should drive the dimer‐tetramer equilibrium towards functional tetramer, thus reducing the gap between the rates of SAM accumulation by the wild‐type and mutant *E. coli* MAT proteins. Indeed, the fivefold increase in protein concentrations closed the gap in SAM accumulation rated (Figure 8b), supporting the latter reasoning. These findings suggest that residues participating in salt‐bridge formation across the small interface are not important recognition determinants for chaperonins that fold EcMAT protomers. In addition, we conclude that aggregation associated with the hydrophobicity of the small interface can be alleviated not only by the salt‐bridges that accelerate dimer–dimer assembly but also by the mass action of higher protein concentration that drives equilibrium towards the aggregation‐free tetramer. However, this solution might be far from optimal under the physiologically relevant conditions, since the requirement to produce higher amounts of MAT to overcome the aggregation constraint can be wasteful for the organism, and possibly, harmful to its metabolism.

### 2.4 Dimers of NgMAT undergo substrate‐induced functional tetramerization

**FIGURE 9:**
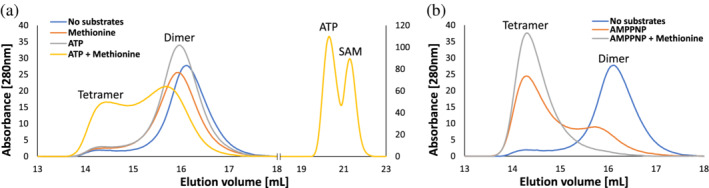
Size exclusion chromatography (SEC) analysis of NgMAT homomeric states in solution in the presence of substrates (a). Addition of both ATP and methionine (yellow trace) markedly increases the fraction of tetrameric species and concomitantly produces S‐adenosylmethionine. (b) Addition of both AMP‐PNP and methionine (gray trace) shifts the distribution entirely toward tetrameric species

As detailed above, analysis of the available crystal structures shows that NgMAT homodimers tend to assemble into tetramers and that the size of the interdimeric interfaces within NgMAT homotetramers is comparable to that of EcMAT (Tables 1 and S1). Yet, in solution, NgMAT assumes a predominantly dimeric state, and the fraction of the tetrameric species remains low even at high protein concentrations (Figure 3b). Moreover, we found that the catalytic turnover (*k*_cat_) of NgMAT was independent of changes in enzyme concentration, suggesting that concentration‐induced shift in dimer–tetramer equilibrium does not affect the catalytic activity (see Section 4 and Figure S13). What is then the functional homomeric state of NgMAT? To address this question, we pre‐incubated 10 μM of NgMAT protein with saturated amounts of methionine, ATP, or a combination of both and analyzed the homomeric states of the samples by size exclusion chromatography (see Section 4). Addition of either substrates alone had no measurable effect on the homomeric state of NgMAT, which remained predominantly dimeric (Figure 9a). However, in the presence of both substrates we saw a marked shift of NgMAT toward tetramerization accompanied by accumulation of the reaction product, SAM (Figure 9a). This suggests that the homotetramer is the functional unit of NgMAT. Next, we repeated the experiment but replaced ATP with a hydrolysis‐resistant analog AMP‐PNP (see Section 4). This analog inhibits the final step of SAM formation, which, in turn, slows the rate of SAM release from the active site.^56^ We found that AMP‐PNP increased the tetrameric species substantially, while in the presence of both AMP‐PNP and methionine the NgMAT protein has shifted almost entirely to a tetrameric state, again indicating that the functional unit of NgMAT is a tetramer (Figure 9b). These findings suggest that NgMAT undergoes substrate‐induced oligomerization into a functional homotetrameric state. If the homotetrameric complex of NgMAT indeed corresponds to its functional state, why does an increase in protein concentration, which is expected to drive the equilibrium toward a functional tetramer, not accompanied by an increase in the apparent *k*_cat_? We, therefore, analyzed the available NgMAT structures. Unfortunately, dimeric NgMAT structures do not exist, so it is not possible to directly observe the structural changes within the interdimeric interface of NgMAT upon substrate‐induced tetramerization. However, we noticed that within the available structures, not all loops gating access to the active sit (residues 95–119) are in closed positions. The movement of these loops is part of the catalytic cycle of MATs; a closed state accompanies SAM formation, while an open state—SAM dissociation.^38^, ^56^ Strikingly, in NgMAT the open state of the loop is accompanied by a dramatic change in the geometry of the small interface. Specifically, opening of the loop moves the side chain of Gln98 by 5.3 Å out of the interface plane relative to the closed state (Figure S14). It is plausible that in the absence of both substrates the active site loops remain in a highly dynamic, predominantly open state(s) that perturb dimer–dimer association, even when the concentration of NgMAT dimers is high. However, in the presence of both ATP and methionine the active site loops tend to assume a rigid closed state, thus removing the entropic barrier for homotetramerization. Thus, similarly to EcMAT, the functional unit of NgMAT is a tetramer. Yet, constraints operating on the folding and assembly of the functional homotetrameric NgMAT state are dramatically different.

## 3 DISCUSSION

A significant fraction (at least half) of the proteins with known structures tends to interact with identical copies of themselves and assemble into homomeric complexes.^4^, ^9^, ^40^ Homomerization provides numerous advantages, including formation of novel active sites, binding of allosteric regulators within interfaces that are remote from active sites, and functional regulation through cooperative substrate binding.^9^, ^14^, ^57^, ^58^, ^59^ It is thus not surprising that homomers are intuitively assumed to be subject to intense evolutionary pressure toward functional diversification. However, there are also strong arguments against the assumption that quaternary homomeric complex formation is a direct result of functional adaptation. Since quaternary structure of homomers does not scale up with organismal complexity or effective population size, it has been argued that homomerization does not necessarily need to be driven by functional adaptation and might instead be a result of stochastic (neutral) forces.^60^, ^61^ Alternatively, homomerization has been suggested to emerge purely as a function‐unrelated side‐effect of thermodynamic stabilization.^62^ However, notwithstanding the original neutral forces of homomerization, homomers can (at least potentially) be recruited by adaptive evolution and undergo functional adaptation at later stages.

Our understanding of the physicochemical and structural determinants that drive and constrain the evolution of homomeric protein structures remains lacking. Here we addressed this problem by analyzing bacterial homologues of MATs, an essential homotetrameric enzyme of central metabolism. Similar to many other core metabolic enzymes,^9^ bacterial MATs are predominantly dihedral homotetramers (dimers of dimers). Ignoring the protomer folding steps, all known dihedral homotetramers assemble via monomer‐dimer‐tetramer path, with the homodimer being the only accessible assembly intermediate in the path toward homotetramerization.^35^, ^63^ We demonstrate that while the large (dimeric) interface of MAT that harbors the active sites appears evolutionary conserved, the small (interdimeric) interface, whose functional significance is less clear, is subject to intense evolutionary diversification. By applying detailed structural and biophysical analysis and focusing on two representative MATs from *E. coli* (EcMAT) and *N. gonorrhoeae* (NgMAT) we established that the diversity in physicochemical complementarity within these small interfaces is linked to variability in kinetic stability and in modes of activity regulation of the homomeric states.

The small interface of EcMAT is more hydrophobic than that of NgMAT, and contains eight salt bridges, which are absent in NgMAT. Removal of these salt‐bridges from the small interface did not affect the rate of EcMAT tetramer–dimer dissociation, indicating that hydrophobic complementarity is sufficient to render the EcMAT homotetramer kinetically stable. However, kinetic stabilization also incurred severe constraints on homotetramer assembly. First, the high activation energy barrier between the assembled tetramer and the transition state along the dimer‐to‐tetramer assembly step imposes a kinetic constraint on the rate of dimer–dimer interactions. Indeed, we found that in the absence of salt‐bridges in the small interface, dimer–dimer association slows down by more than an order of magnitude (Figure 7a–c). Second, in the absence of salt‐bridges, the dimeric assembly intermediate is highly prone to aggregation (Figure 7d–f). Our data suggest, therefore, that the role of the salt‐bridges that are found in the EcMAT small interface but absent in all other known crystal structures of bacterial MATs (Table 1), is to alleviate the kinetic and aggregation constrains imposed by the hydrophobicity of the small interface on the dimeric assembly intermediate. The long‐range electrostatic interactions accelerate dimer‐to‐tetramer assembly, thus reducing the time that aggregation‐prone dimers are lingering unassembled. It is plausible that the high hydrophobicity of the small interface constraints not only the dimer‐to‐tetramer assembly step but also folding of individual MAT protomers by rendering them aggregation‐prone. Therefore, the obligatory folding dependency of EcMAT on GroEL/ES chaperonins might be the result of such a constraint. Since MATs from *N. gonorrhoeae* and *L. plantarum* fold and assemble in the absence of chaperonins (Figure 6), the folding dependency of EcMAT is not a general inherent property of the MAT fold. We note that sustaining an obligatory chaperonin dependency inexorably imposes a fitness cost on the organism. The dependency is not only energetically costly (one refolding cycle by GroEL/ES burns 14 ATP molecules^64^), but it also diverts chaperonins, an essential and limited resource, from other proteins that might require folding assistance, particularly under proteostatic stress. Thus, one would anticipate that purifying selection will operate to wean proteins off folding dependency on chaperonins, if this dependency is a result of random mutations that jeopardize protein folding and stability. We, therefore, propose that the obligatory EcMAT chaperonin dependency might be an outcome of adaptive pressure to kinetically stabilize MAT quaternary complexes in *E. coli*. Why would the kinetic stabilization of MAT quaternary structure be advantageous for *E. coli*? Thermodynamic and kinetic stabilities are crucial parameters that determine the intracellular turnover of proteins in bacteria.^65^, ^66^, ^67^ Since protein degradation in vivo occurs through proteolysis,^68^ a high free energy barrier for protein complex dissociation will prolong its intracellular half‐life, simply because protein states beyond the barrier (i.e., assembly intermediates) are more vulnerable to proteolytic attack.^69^, ^70^, ^71^ Kinetic stabilization of proteins might be particularly important in bacteria like *E. coli* that are subject to prolonged starvation periods accompanied by starvation‐induced proteome degradation.^72^, ^73^, ^74^, ^75^

In striking contrast to EcMAT, the small interface of NgMAT is less hydrophobic and lacks salt‐bridges (Table 1). As a result, the constraints imposed by the small interface in EcMAT on protomer folding and complex assembly are absent in NgMAT. In the absence of reaction substrates, NgMAT adopts a predominantly dimeric state in solution, whose kinetic stability is significantly lower than that of the tetrameric EcMAT (Figure 5b). The rate of NgMAT urea‐induced disassembly/unfolding appears to be independent of protein concentration (Figure 5c), suggesting that these two processes are coupled. Furthermore, NgMAT protomer folding and dimerization might also be coupled. Association of unstructured (or partially structured) monomers followed by dimer folding has been reported for several proteins, including a homotetrameric p53.^76^, ^77^ The idea that NgMAT dimerization and protomer folding might be coupled is also supported by the fact that NgMAT folding and assembly appears to be independent of chaperonins (Figure 6). Indeed, GroEL chamber can accommodate polypeptides smaller than 60 kDa, whereas the MAT mobomer is approximately 42 kDa. Thus, MAT protomers whose folding is chaperonin‐dependent, are expected to assume a folded state prior to dimerization, precluding the possibility for a coupled folding/assembly step. Another novel feature of NgMAT homomerization, is that unlike EcMAT dimers that rapidly assemble into tetramer, NgMAT dimers “resist” tetramerization even at high protein concentration. The dimeric state is presumably preferred because of the high flexibility of the active site loops and the relatively low hydrophobicity of the interface. In the absence of substrates, the loop movement perturbs the small interface, thus generating a high activation energy barrier for tetramerization. Strikingly, only upon addition of both reaction substrates, methionine and ATP, does the functional tetramer assembly. When both substrates are present, the flexible loops that guard the active sites assume a closed conformation, thus removing the structural hindrance for dimer–dimer association. Importantly, this dominant effect of the active site loop conformations on the binding affinity of the NgMAT small interface effectively prevents the possibility for regulation of the NgMAT homotetramerization (and, thus, NgMAT activity) by tuning its intracellular concentration. Assuming that the intracellular levels of NgMAT are similar to those established for EcMAT (600 nM),^78^ the upregulation of the protein expression by the cell even by over 160‐fold (up to 100 μM) will not shift significantly the dimeric fraction toward functional tetrameric species (Figure 4b). Thus, it appears that the structural mechanism that has emerged to ensure an effective substrate‐induced regulation of functional tetramerization of NgMAT has also led to a minimization of the protein concentration‐dependent tetramerization. Another important insight from the detailed analysis of NgMAT homomeric states is that neither the oligomeric state of the protein in the crystal (a tetramer, PDB ID 5T8S) nor the biological assembly assignment of this structure based on the prediction of the free energy of complex disassembly (a dimer) reveal the true complexity of NgMAT behavior in solution: dimer that undergoes substrate‐induced tetramerization.

Our findings therefore suggest that evolution recruited the interdimeric interface of bacterial MATs to tune the properties of MAT complexes to the environmental and metabolic needs of bacteria. The evolutionary advantage to regulate the properties of MAT quaternary complexes via the small interface is clear—it allows evolutionary plasticity far from the crucial active sites, which are subject to severe evolutionary pressure to preserve function. The evolutionary diversification of the small interface thereby produced MAT complexes with unique properties, but this process also imposed unique constraints on MAT folding and assembly.

## 4 METHODS

### 4.1 Interface analysis and visualization

Interfaces of various MATs were characterized using PISA server.^43^ The interface area is calculated by PISA as a difference in total accessible surface areas of isolated and interfacing structures divided by two. Change in the solvation free energy upon formation of the interface, *Δ*G_i_ in kcal/mol, is calculated as a difference in total solvation energies of isolated and interfacing structures. Negative *Δ*G_i_ corresponds to hydrophobic interfaces and does not include the effect of satisfied hydrogen bonds and salt bridges across the interface. PISA also predicts hydrogen‐bond and salt‐bridge formation in the interface. All parameters pertaining to the inter‐dimeric interface are presented as summation over four individual patches (Table 1). Residues participating in interface interactions were visualized as spheres using PyMol, colored according to per‐residue hydrophobicity.^44^

### 4.2 Gene cloning, protein expression, and purification

The gene encoding NgMAT was custom synthesized by Integrated DNA Technologies with a fused fragment encoding C‐terminal Hisx6 tag and flanking NdeI and XhoI restriction sites. To this end, the protein sequence of NgMAT (NCBI Reference Sequence: WP_003687370.1) was converted to DNA sequence using the manufacturer's *E. coli* codon optimization tool. The gene encoding LpMAT was amplified directly from the chromosome of *Lactobacillus plantarum*, using the following primers for Gibson assembly:

For

AATTCCCCTCTAGAAATAATTTTGTTTAACTTTAAGAAGGAGATATACATATGAGTGAAAGACACTTATTTACATCTGAATCTGTCTCTG.

and *Rev*.

AGCCAACTCAGCTTCCTTTCGGGCTTTGTTAGCAGCCGGATCTTAATGGTGATGGTGATGGTGTTTAAATGCTGCTTTTAGGGCATCCAC. EcMAT gene was cloned as in Reference 11. EcMATmut (E67K and K98Q) and NgMATmut (K67E and Q98K) were generated by site directed mutagenesis. All MAT genes were cloned into pET24a expression system and expressed in BL21 (DE3) cells. Specifically, an overnight starter was diluted 1:100, grown at 37°C until OD at 600 nm was 0.5, after which the expression was induced by the addition of 0.4 mM IPTG overnight at 30°C. Cells were centrifuged at 4800×g, and the pellet was stored at −20°C. Cells were lysed by sonication after a 30‐min preincubation with 1 mg/ml lysozyme (Merck, Germany) and 500 U benzonase (Merck, Germany) on ice. The filtered lysate was purified by Ni‐NTA on a His‐TRAP FF 5‐ml column (GE Healthcare, US) and dialyzed into 25 mM Tris pH 8.0, 1 mM DTT, 50% glycerol. Denatured MATs were purified on the same column, according to manufacturer instructions. Briefly, pellets were lysed in 100 mM NaH_2_PO_4_, 10 mM TrisHCl, 6 M GuHCl adjusted to pH 8.0, and loaded on the column. Two washes were performed using the same buffer, lowering the pH to 6.3 and later to 5.9. Finally, the protein was eluted at a pH of 4.5. Using far‐UV CD analysis we validated that the obtained denatured proteins are void of secondary structure (see Section 4 section “Denaturant‐induced equilibrium unfolding” and Figure S4d–f). The strain TG1/PoA GroEL + GroES was graciously gifted to us by the lab of Amnon Horwitz and was used to overexpress *E. coli* GroEL and Hisx6‐tagged GroES, using the protocol described above. The filtered lysate was purified by Ni‐NTA on a His‐TRAP FF 5‐ml column (GE Healthcare, US), collecting the flow through (containing the overexpressed GroEL) and the eluted fractions (containing purified GroES). The fractions were dialyzed against 25 mM TrisHCl pH 8.0, 150 mM KCl, 1 mM DTT and flash frozen as aliquots and stored at −80°C. The flow through incubated at 60°C for 7 min, centrifuged at 4800×g, n treated with 67% ammonium sulfate and then centrifuged again (4800×g). Pellet was resuspended in 20 ml 25 mM TrisHCl pH 8.0, 1 mM DTT and treated with a dropwise addition of acetone with rapid stirring up to a final concentration of 45%. The resulting mixture was centrifuged at 4800×g, followed by the dialysis of the supernatant against 25 mM TrisHCl pH 8.0, 1 mM DTT. The protein was then purified by size exclusion chromatography using a superpose six column and concentrated using centrifuge filters Amicon Ultra, Merck, Germany. Aliquots were flash frozen and stored at −80°C.

### 4.3 In vivo assay to assess the MAT dependency on GroEL/ES

The assay is a modification of a previously published approach.^48^ Briefly, MGM100 *E. coli* strain with arabinose‐inducible expression of groL/S genes^79^ was transformed with pFLAG expression plasmid (Merck, Germany) carrying a MAT gene (from either *E. coli*, *U. urealyticum*, *L. plantarum*, or *N. gonorrhoeae*). Strains were grown on LB supplemented with 0.2% arabinose, at 37°C to log phase, after which they were centrifuged and washed with fresh LB. The cells were then diluted into LB with 1 mM diaminopimelate, with either 0.2% arabinose or 0.2% glucose. Dilution ratios were 1:5,000 for the arabinose condition and 1:500 for the glucose condition. The cells were harvested 5.5 hr after growth at 37°C. MAT dependency was assessed by (i) a drop in soluble MAT levels, visualized by Western blot using custom anti‐MAT antibodies, and (ii) by overexpression of the MetE protein, which is activated when intracellular SAM levels are low.^54^

### 4.4 Analytical SEC

Size exclusion chromatography was performed on Superdex 200 Increase 30 × 1 cm column, equilibrated with 25 mM TrisHCl, 150 mM KCl, 1 mM DTT. MATs were injected at a monomeric concentration of 10 μM, unless specified otherwise. Molecular weight of proteins was determined by a calibration curve (Figure S6) using the following as standards: Albumin, Cytochrome C, Beta Amylase, Carbonic Anhydrase and Alcohol Dehydrogenase.

### 4.5 *K*_d_ calculation

The fraction (F) of tetramers present at dimer‐tetramer equilibrium can be expressed by:

(1) $$F=\frac{\left[T\right]}{\left[\text{total protein}\right]}=\frac{\left[T\right]}{\left[D\right]+\left[T\right]},$$

where [T] and [D] are concentrations of tetramers and dimers, respectively. The dissociation constant (*K*_d_) can be expressed by:

(2) $$K_{d}=\frac{{\left[D\right]}^{2}}{\left[T\right]},$$

which can be reorganized to give:

(3) $$\left[T\right]=\frac{{\left[D\right]}^{2}}{K_{d}}.$$

Combining Equation (1) and Equation (3) we get:

$$F=\frac{{\left[D\right]}^{2}}{K_{d}\left(\left[D\right]+\frac{{\left[D\right]}^{2}}{K_{d}}\right)}=\frac{\left[D\right]}{K_{d}\left(1+\frac{\left[D\right]}{K_{d}}\right)}=\frac{\left[D\right]}{K_{d}+\left[D\right]},$$

which can be further reorganized to give:

(4) $$K_{d}=\frac{\left[D\right]}{F}-\left[D\right],$$

By substituting the values for fraction of tetramers in equilibrium from Figure 7b,c, and calculating the dimer concentration as half the monomeric concentration of nontetrameric fraction, we arrive at:

$${K_{d}}^{\mathrm{WT}}=\frac{3.5\mu M}{0.3}-3.5\;\mu M=8.17\;\mu M\;\text{and}\;{K_{d}}^{\mathrm{mut}}=\frac{4.75\mu M}{0.05}-4.75\;\mu M=90.25\;\mu M.$$

Thus, the ratio between the two dissociation constants is 11.05.

### 4.6 Protein crystallization, data collection, structure determination, and refinement

PDB ID# 7R2W. 6.55 mg/ml EcMATmut +5 mM AMPPNP +5 mM methionine were mixed 1:1 (v/v) with reservoir solution, and crystallized by the sitting drop vapor diffusion method over a reservoir containing 0.2 M L‐Proline, 0.1 M Hepes pH 7.56, and 14% PEG 3350 at room temperature. Crystals were harvested, cryoprotected, and flash‐cooled in liquid N_2_. X‐ray diffraction data were collected at beamline IO3 of Diamond Light Source (Didcot, UK). Data were collected at 100 K from one crystal of EcMATmut+AMPPNP+Met that diffracted to a maximum resolution of 1.6 Å. The obtained EcMATmut +AMPPNP+Met crystal belongs to the space group P42212, with unit cell dimensions a 86.6113, b 86.6113, and c 90.9818, and it contained one copy of the protein in the asymmetric unit. X‐ray data were merged and scaled using an automatic pipeline of CCP4 cloud, and the structure was solved by molecular replacement using Phaser^80^ in CCP4 cloud.^81^ Ensemble of monomers of MATs from different sources were set as a search model. Refinement employed alternating cycles of manual rebuilding in COOT^82^ and automated refinement using Refmac5^83^ in CCP4 cloud. The coordinates and structure factors have been submitted to the PDB under the accession code 7R2W.

PDB ID# 7R3B. 3.8 mg/ml LpMAT +5 mM AMPPNP +5 mM methionine were mixed 1:1 (v/v) with reservoir solution, and crystallized by the sitting drop vapor diffusion method over a reservoir containing 50 mM CaCl_2_, 0.1 M Bis‐Tris pH 6.8, 32.14% PEG 550 MME at room temperature. Crystals were harvested, cryoprotected, and flash‐cooled in liquid N_2_. X‐ray diffraction data were collected at beamline IO4 of Diamond Light Source (Didcot, UK). Data were collected at 100 K from one crystal of LpMAT+AMPPNP+Met that diffracted to a maximum resolution of 2.82 Å. The of LpMAT+AMPPNP+Met crystal belongs to the space group P1, with unit cell dimensions a 58.43, b 110.93, and c 112.66, and it contained eight copies of the protein in the asymmetric unit. X‐ray data were merged and scaled using XDS^84^ and was solved by molecular replacement using Phaser^80^ in CCP4.^81^ Native monomer of MAT from *E. coli* (PDB ID: 1FUG) was used as a search model. Refinement employed alternating cycles of manual rebuilding in COOT^82^ and automated refinement using Refmac5.^83^ The coordinates and structure factors have been submitted to the PDB under the accession code 7R3B.

### 4.7 Urea‐induced dissociation kinetics

EcMAT and NgMAT samples (6 and 30 μM), as well as EcMATmut (30 μM) were prepared in 25 mM TrisHCl pH 8.0, 150 mM KCl, 1 mM DTT. The dissociation kinetics were measured in SX20 stopped‐flow (Applied Photosystems, UK) by mixing the protein in 1:5 ratio with 7.2 M urea (6 M urea final concentration) prepared in the same buffer. The ensuing perturbation in tryptophan fluorescence (ex. 295 nm, em. 343 nm) was measured, and the obtained signal was fitted to a single exponential:

$$A\left(t\right)-A\left(\infty \right)=\sum A_{i}\times e\left(-k_{i}t\right),$$

where *A*(*t*) is the amplitude at time *t*, *A*(∞) is the offset value, *A*_*i*_ is the change in signal for phase *i*, and *k*_*i*_ is the observed rate at phase *i*.

### 4.8 Enzymatic activity

Native MAT activity (250 nM) was determined at 37°C in activity buffer (25 mM Tris–HCl pH 8.0, 100 mM KCl, 10 mM MgCl_2_, 1 mM DTT) at saturated ATP and methionine concentrations (5 mM). Reaction initiation, sample collection and analysis were performed as in Reference 11. Rate of accumulation of SAM upon refolding of GuHCl‐unfolded EcMAT and EcMATmut was determined at 250 nM unfolded MAT at 37°C in the activity buffer, with the addition of 20 mM ATP and 5 mM methionine. For refolding to take place, the reaction occurred in the presence of 1:2 and 1:4 M excess (monomer MAT to complete system) of GroEL and GroES, respectively. To eliminate the risk of running out of ATP, an ATP regeneration system (40 mM PEP, 40 Units/ml PK) was also added to the mix. Reaction sample collection and analysis was performed as above.

### 4.9 Denaturant‐induced equilibrium unfolding

EcMAT and NgMAT samples (10 μM) were incubated in 25 mM TrisHCl pH 8.0, 150 mM KCl, and 1 mM DTT in the presence of various urea/GuHCl concentrations (0–6 M), at room temperature for 24 hr. To determine the fraction of secondary structure, circular dichroism (CD) spectra of each sample was measured in the range of 210–260 nm, using Jasco J‐815 CD spectrometer (Figure S4).

### 4.10 Energy calculations of residue‐level contributions to interactions across the small interface

The three‐dimensional structures of EcMAT (PDB #ID 1P7L), EcMATmut (PDB #ID 7R2W, this work) and NgMAT (PDB #ID 5T8S) were used in our energy calculations. Hydrogen atoms were added using CHARMM and the structures were subjected to conjugate gradient minimization with a harmonic restraint force of 50 kcal/mol/Å^2^ applied to the heavy atoms. Energy calculations to analyze per‐residue contributions were performed following the methodology described previously.^45^, ^85^, ^86^ The Finite Difference Poisson–Boltzmann method, as implemented in DelPhi,^87^ was used to calculate the net electrostatic/polar contributions (ΔΔ*G*_elec_) of each residue within 15 Å of the small interface in each tetramer complex. For each residue, electrostatic contributions from each side chain or the entire residue were calculated separately, and comparison of these separate calculations was used to determine if electrostatic contributions originate from the side‐chain of a residue, the main chain, or both. Residues contributing ΔΔ*G*_elec_ ≥ 1 kcal/mol to the interactions (twice the numerical error of the electrostatic calculations) were deemed as substantially contributing to the interactions.^45^ Nonpolar energy contributions (ΔΔ*G*_np_) were calculated as a surface‐area proportional term by multiplying the per‐residue surface area buried upon complex formation, calculated using Surfv,^88^ by a surface tension constant of 0.05 kcal/mol/Å^2^.^45^ Residues contributing ΔΔ*G*_np_ ≥ 0.5 kcal/mol to the interactions (viz those that bury more than 10 Å^2^ of each protein surface upon complex formation) were defined as making substantial nonpolar contributions. Contributions specific to the small interface between tetramer subunits were determined by comparing the calculated contributions in the tetramer to those calculated in a trimer structure (i.e., a single chain vs. a dimer). To reduce false positives and negatives, we applied a consensus approach across the four chains of each tetramer.

## AUTHOR CONTRIBUTIONS

**Daniel Kleiner:** Data curation (lead); formal analysis (lead); investigation (lead); visualization (lead). **Ziva Shapiro Tuchman:** Formal analysis (supporting); visualization (supporting). **Fannia Shmulevich:** Data curation (supporting); project administration (supporting). **Anat Shahar:** Data curation (supporting); formal analysis (supporting); validation (supporting). **Raz Zarivach:** Data curation (supporting); formal analysis (supporting); validation (supporting). **Mickey Kosloff:** Data curation (supporting); formal analysis (supporting); writing – review and editing (supporting). **Shimon Bershtein:** Conceptualization (lead); funding acquisition (lead); methodology (lead); resources (lead); supervision (lead); writing – original draft (lead).

## Supporting information


**Figure S1**. Crystal structure of MAT from *L. planatrum*
**Figure S2**. Surface representation of the large (dimeric) interfaces of bacterial MATs
**Figure S3**. Structural comparison of MATs from *N. gonorrhoeae* and *E. coli*
**Figure S4**. Denaturant‐induced equilibrium unfolding of MATs from *N. gonorrhoeae* and *E. coli*
**Figure S5**. Residue‐level structure‐based energy calculations
**Figure S6**. Calibration curve for size exclusion chromatography
**Figure S7**. Size exclusion chromatography (SEC) analysis of LpMAT
**Figure S8**. Removal of salt‐bridge forming residues in EcMAT does not affect catalytic activity.
**Figure S9**. Size exclusion chromatography (SEC) analysis of NgMAT K67E Q98K.
**Figure S10**. Crystal structure of EcMAT mutant
**Figure S11**. Stopped‐flow kinetics of the urea‐induced apparent dissociation/unfolding rate
**Figure S12**. Folding/assembly of EcMAT and EcMATmut is chaperonin‐dependent in vitro
**Figure S13**. Catalytic turnover of EcMAT and NgMAT is concentration independent
**Figure S14**. Movement of the active site loops in NgMAT

**Table S1** List of available bacterial MAT crystal structures

**Table S2** Crystallographic data collection and refinement statistics
