# Supplementary material for: Evolution of homo‐oligomerization of methionine S‐adenosyltransferases is replete with structure–function constrains
Source: Protein Sci. 2022 Jun 16;31(7):e4352. doi: 10.1002/pro.4352 (PMC9202080; doi:10.1002/pro.4352)
Supplement: Supplementary file 1 — Figure S1. Crystal structure of MAT from L. planatrum Figure S2. Surface representation of the large (dimeric) interfaces of bacterial MATs Figure S3. Structural comparison of MATs from N. gonorrhoeae and E. coli Figure S4. Denaturant‐induced equilibrium unfolding of MATs from N. gonorrhoeae and E. coli Figure S5. Residue‐level structure‐based energy calculations Figure S6. Calibration curve for size exclusion chromatography Figure S7. Size exclusion chromatography (SEC) analysis of LpMAT Figure S8. Removal of salt‐bridge forming residues in EcMAT does not affect catalytic activity. Figure S9. Size exclusion chromatography (SEC) analysis of NgMAT K67E Q98K. Figure S10. Crystal structure of EcMAT mutant Figure S11. Stopped‐flow kinetics of the urea‐induced apparent dissociation/unfolding rate Figure S12. Folding/assembly of EcMAT and EcMATmut is chaperonin‐dependent in vitro Figure S13. Catalytic turnover of EcMAT and NgMAT is concentration independent Figure S14. Movement of the active site loops in NgMAT [file PRO-31-e4352-s001.pdf]

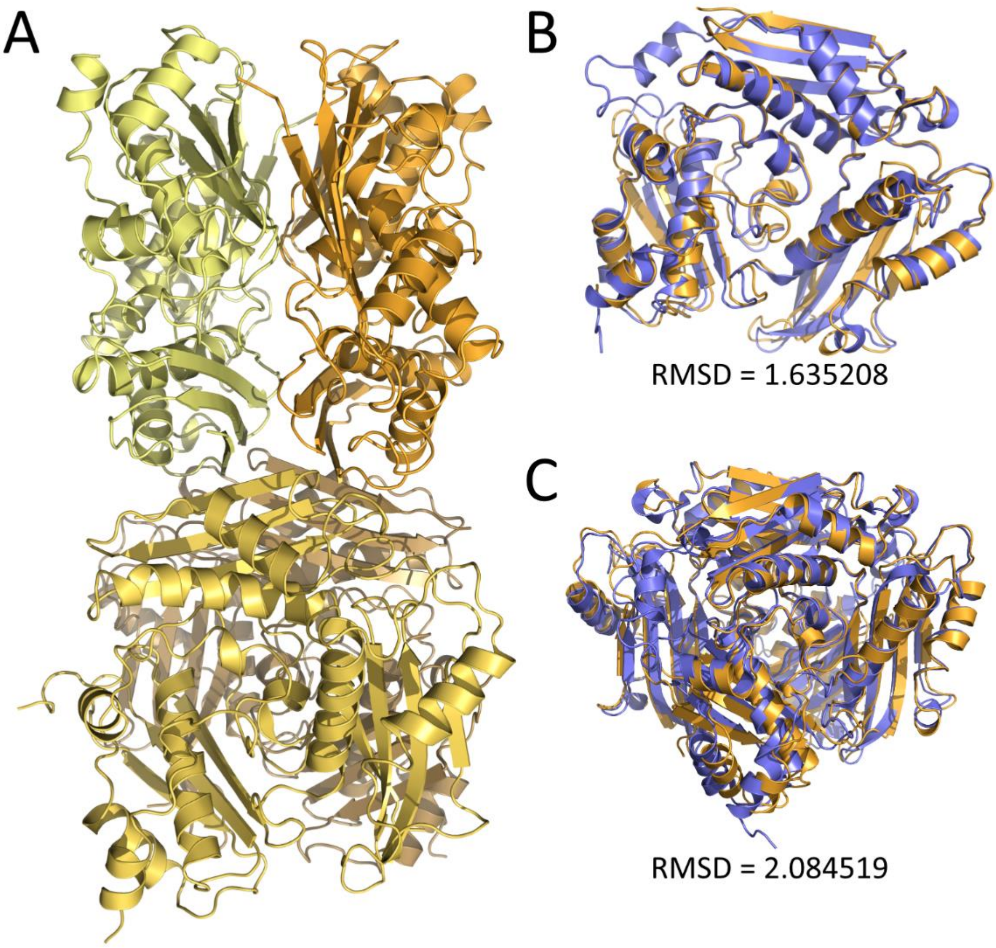

Figure S1. Crystal structure of MAT from *L. planatrum*. A. Carton representation of LpMAT homotetramer. Individual subunits are shown in different shades of yellow and orange. Structural alignment of EcMAT (purple) and LpMAT (orange) monomers **(B)** and dimers **(C)** shows high structural conservation. RMSD, root-mean-square deviation.

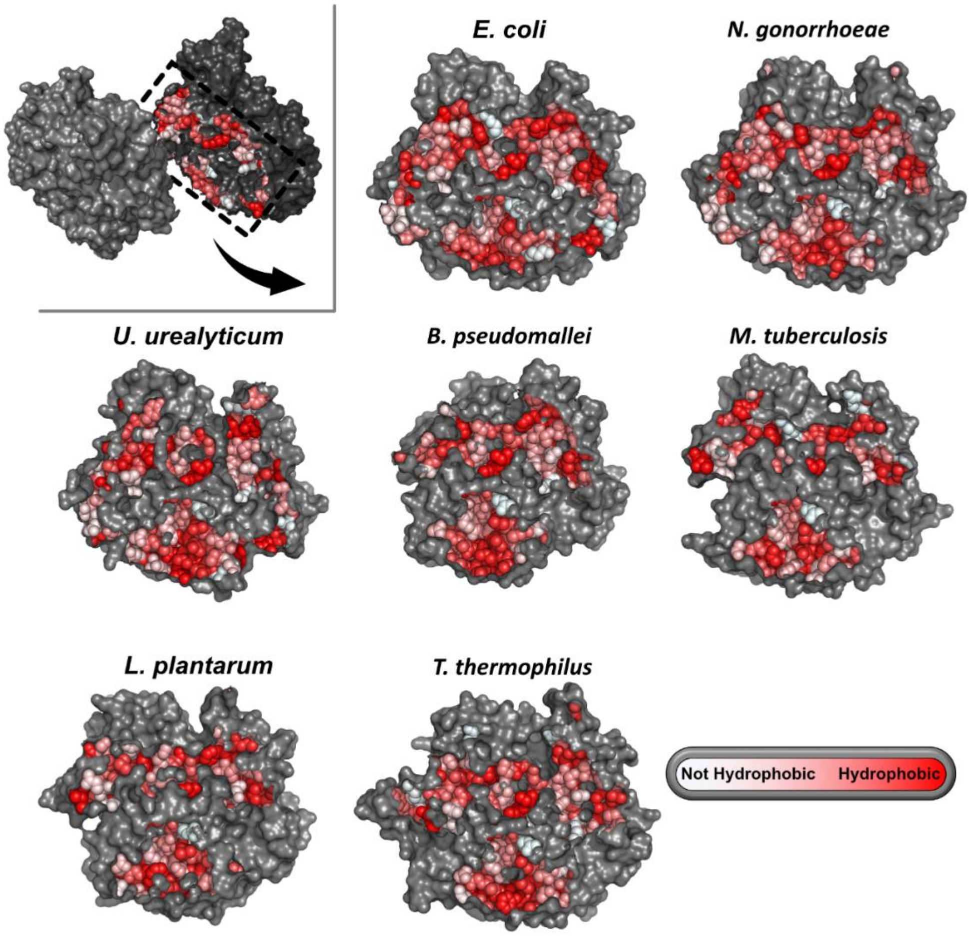

Figure S2. Surface representation of the large (dimeric) interfaces of bacterial MATs. Residues directly forming the dimer-dimer interaction are colored according to their hydrophobicity.

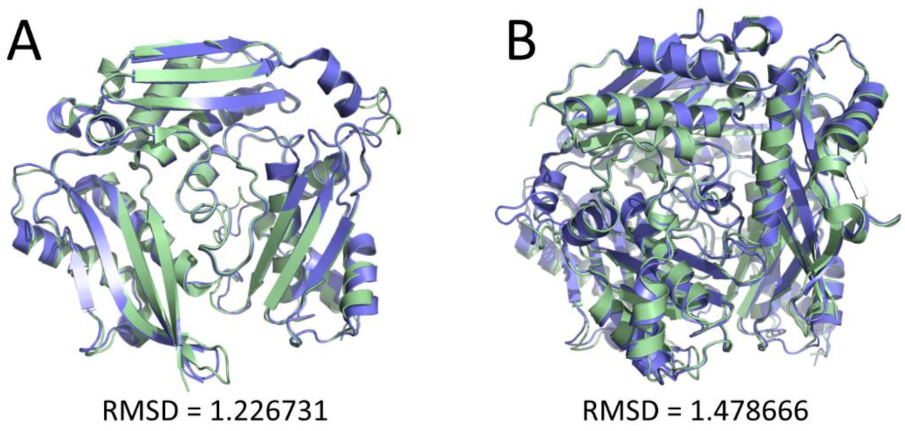

Figure S3. Structural comparison of MATs from *N. gonorrhoeae* and *E. coli*. A. Structural alignment of EcMAT (purple) and NgMAT (green) monomers **(A)** and dimers **(B)** shows high structural conservation. RMSD, root-mean-square deviation.

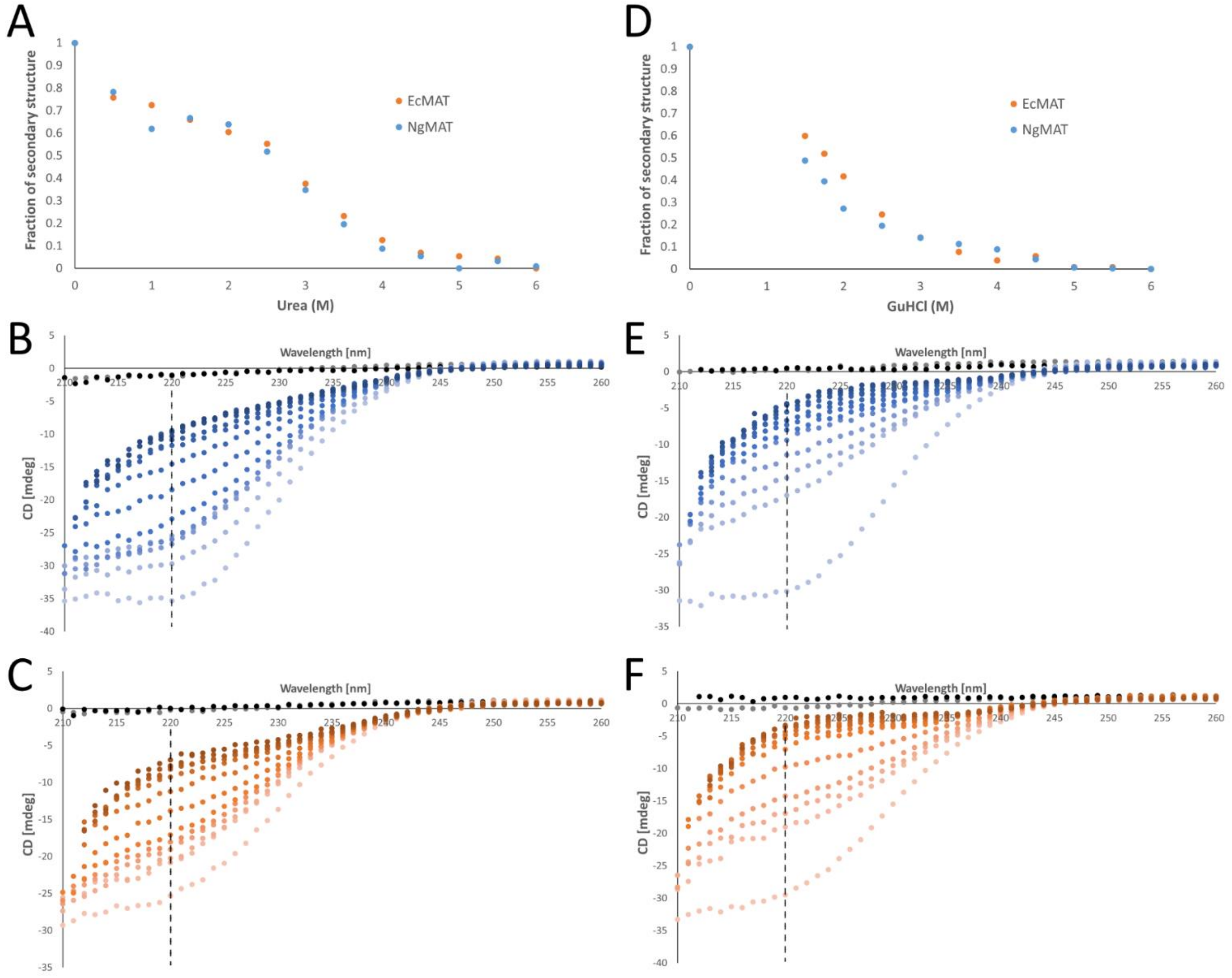

Figure S4. Denaturant-induced equilibrium unfolding of MATs from *N. gonorrhoeae* and *E. coli*. Fraction of residual MAT secondary structure in the presence of urea **(A)** or GuHCl **(D)**, based on CD measurements at 220nm (dashed lines in **B**,**C**,**E**,**F**). Full CD spectra (210-260nm) of NgMAT (blue, **B** and **E**) and EcMAT (orange, **C** and **F**), as a function of increasing urea (**B** and **C**), or GuHCl (**E** and **F**) concentration (0-6M, light color to dark color, respectively). Control samples containing buffer only are shown as gray (buffer with 0M denaturant) and black (buffer with 6M denaturant) dots.

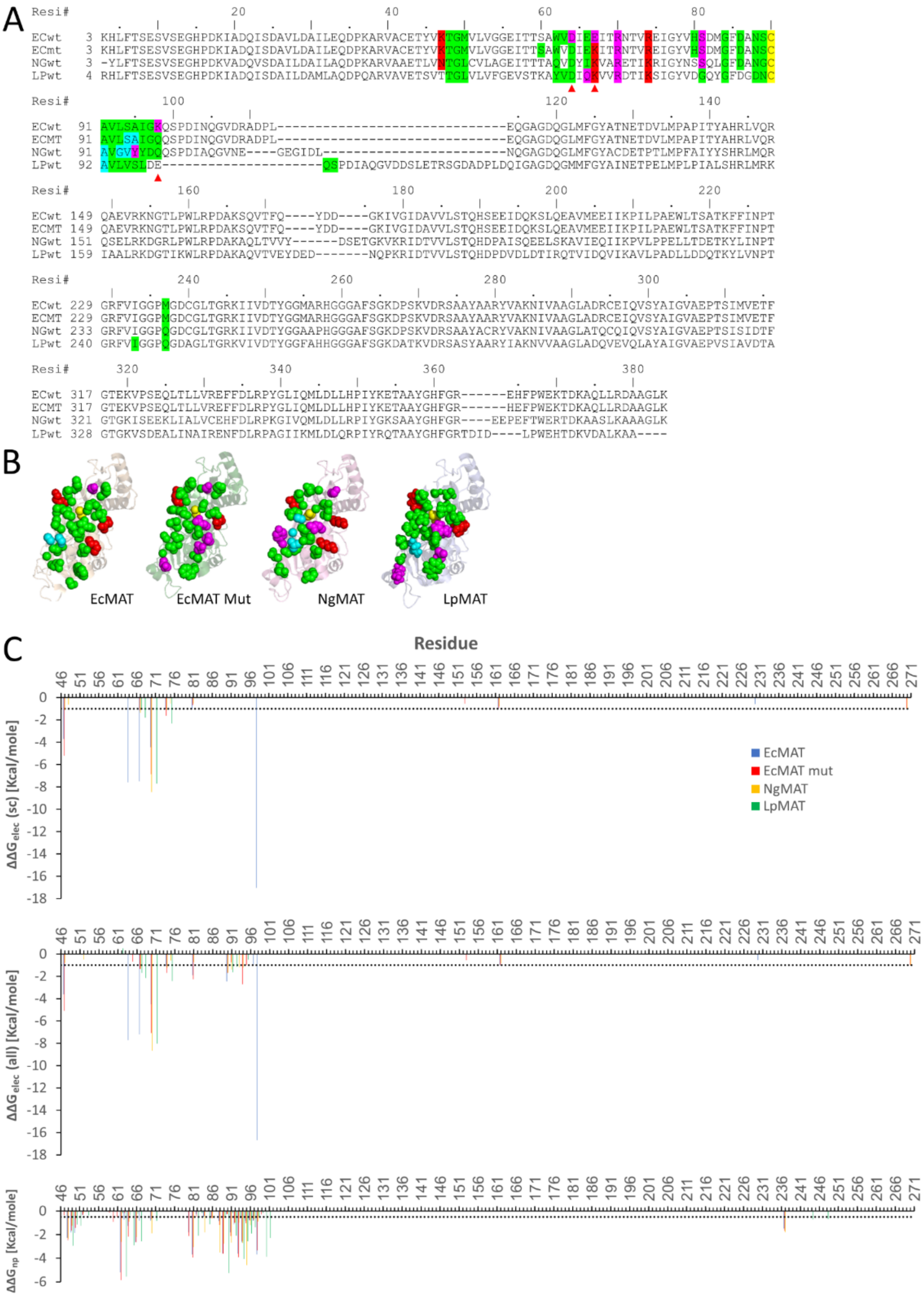
A
Resi# 20 40 60 80
ECwt 3 KHLFTSESVSEGHPDKIADQISDAVLDAILEQDPKARVACETYVKTGMVLVGGEITTSAWVDIEEITRNTVREIGYVHSDMGFDANSC
ECmt 3 KHLFTSESVSEGHPDKIADQISDAVLDAILEQDPKARVACETYVKTGMVLVGGEITTSAWVDIEKITRNTVREIGYVHSDMGFDANSC
NGwt 3 -YLFTSESVSEGHPDKVADQVSDAILDAILAQDPKARVAAETLVNTGLCVLAGEITTTAQVDYIKVARETIKRIGYNSSQLGFDANGC
LPwt 4 RHLFTSESVSEGHPDKIADQISDAILDAMLAQDPQARVAVETSVTTGLVLVFGEVSTKAYVDIQKVVRDTIKSIGYVDGQYGFDGDNC
Resi# 100 120 140
ECwt 91 AVLSAIGKQSPDINQGVDRADPL------------------------------EQGAGDQGLMFGYATNETDVLMPAPITYAHRLVQR
ECMT 91 AVLSAIGQQSPDINQGVDRADPL------------------------------EQGAGDQGLMFGYATNETDVLMPAPITYAHRLVQR
NGwt 91 AVGVYYDQQSPDIAQGVNE----GEGIDL------------------------NQGAGDQGLMFGYACDETPTLMPFAIYYSHRLMQR
LPwt 92 AVLVSLDE---------------------QSPDIAQGVDDSLETRSGDADPLDQIGAGDQGMMFGYAINETPELMPLPIALSHRLMRK
Resi# 160 180 200 220
ECwt 149 QAEVRKNGTLPWLRPDAKSQVTFQ----YDD----GKIVGIDAVVLSTQHSEEIDQKSLQEAVMEEIIKPILPAEWLTSATKFFINPT
ECMT 149 QAEVRKNGTLPWLRPDAKSQVTFQ----YDD----GKIVGIDAVVLSTQHSEEIDQKSLQEAVMEEIIKPILPAEWLTSATKFFINPT
NGwt 151 QSELRKDGRLPWLRPDAKAQLTVVY------DSETGKVKRIDTVVLSTQHDPAISQEELSKAVIEQIIKPVLPPELLTDETKYLINPT
LPwt 159 IAALRKDGTIKWLRPDAKAQVTVEYDED-------NQPKRIDTVVLSTQHDPDVDLDTIRQTVIDQVIKAVLPADLLDDQTKYLVNPT
Resi# 240 260 280 300
ECwt 229 GRFVIGGPMGDCGLTGRKIIVDTYGGMARHGGGAFSGKDPSKVDRSAAYAARYVAKNIVAAGLADRCEIQVSYAIGVAEPTSIMVETF
ECMT 229 GRFVIGGPMGDCGLTGRKIIVDTYGGMARHGGGAFSGKDPSKVDRSAAYAARYVAKNIVAAGLADRCEIQVSYAIGVAEPTSIMVETF
NGwt 233 GRFVIGGPQGDCGLTGRKIIVDTYGGAAPHGGGAFSGKDPSKVDRSAAYACRYVAKNIVAAGLATQCQIQVSYAIGVAEPTSISIDTF
LPwt 240 GRFVIGGPQGDAGLTGRKVIVDTYGGFAHHGGGAFSGKDATKVDRSASYAARYIAKNVVAAGLADQVEVQLAYAIGVAEPVSIAVDTA
Resi# 320 340 360 380
ECwt 317 GTEKVPSEQLTLLVREFFDLRPYGLIQMLDLLHPIYKETAAYGHFGR------EHFPWEKTDKAQLLRDAAGLK
ECMT 317 GTEKVPSEQLTLLVREFFDLRPYGLIQMLDLLHPIYKETAAYGHFGR------HEFPWEKTDKAQLLRDAAGLK
NGwt 321 GTGKISEEKLIALVCEHFDLRPKGIVQMLDLLRPIYGKSAAYGHFGR----EEPEFTWERTDKAASLKAAAGLK
LPwt 328 GTGKVSDEALINAIRENFDLRPAGIIKMLDLQRPIYRQTAAYGHFGRTDID----LPWEHTDKVDALKAA----
B
EcMAT
EcMAT Mut
NgMAT
LpMAT
C
Residue
$\Delta\Delta G_{elec}$ (sc) [Kcal/mole]
$\Delta\Delta G_{elec}$ (all) [Kcal/mole]
$\Delta\Delta G_{np}$ [Kcal/mole]
EcMAT
EcMAT mut
NgMAT
LpMAT

Figure S5. Residue-level structure-based energy calculations for the contributions of a representative monomer in a MAT tetramer across the inter-dimeric interface. A. Full-length amino acid sequence alignment of EcMAT, EcMATmut, NgMAT, and LpMAT. Residue numbering are according to EcMAT. Residues with substantial contribution to intermolecular interactions are colored according to the “type” of their energy contribution: non-polar contributions (np), green; side-chain electrostatic contributions (sc elec), red; main-chain electrostatic contributions, yellow; sc elec and np, magenta; mc elec and np, cyan. The three residues contributing to the formation of the EcMAT salt bridges across the interface (see the main text) are marked with red triangles below the alignment. **B.** 3D representation of residues (spheres) shown in **(A)**, in EcMAT, EcMATmut, NgMAT, and LpMAT – shown as wheat, green, pink ribbons, and grey, respectively. **C.** Per-residue energy contributions to MAT interactions across the inter-dimeric interface in the three different complexes. Panels show the results of the energy calculations for inter-dimeric interactions. The dashed lines mark the -1 kcal/mol threshold for $\Delta\Delta G_{elec}$ and -0.5 kcal/mol threshold for $\Delta\Delta G_{np}$ used to determine substantial contributions to the interaction.

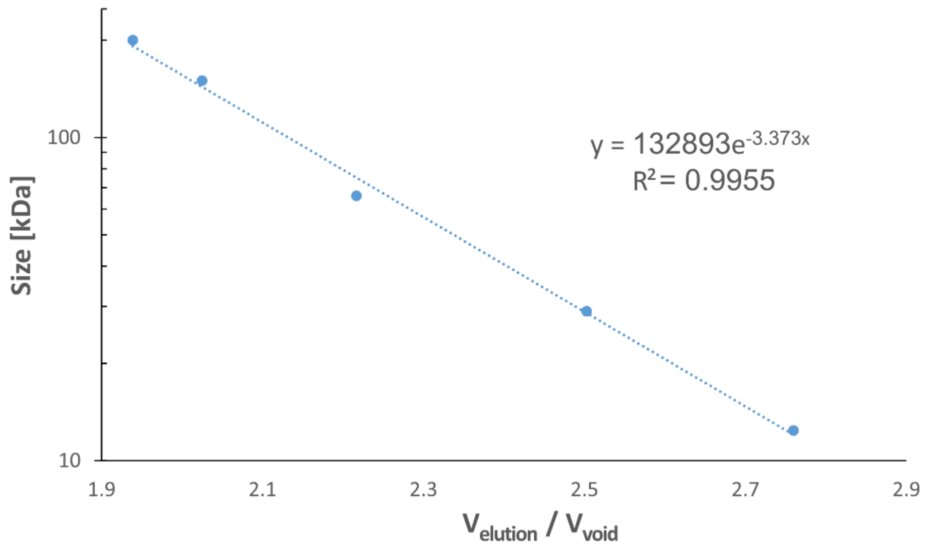

Figure S6. Calibration curve for size exclusion chromatography (SEC). Molecular weight of protein standards (log scale) is plotted against a ratio of elution volume and void volume.

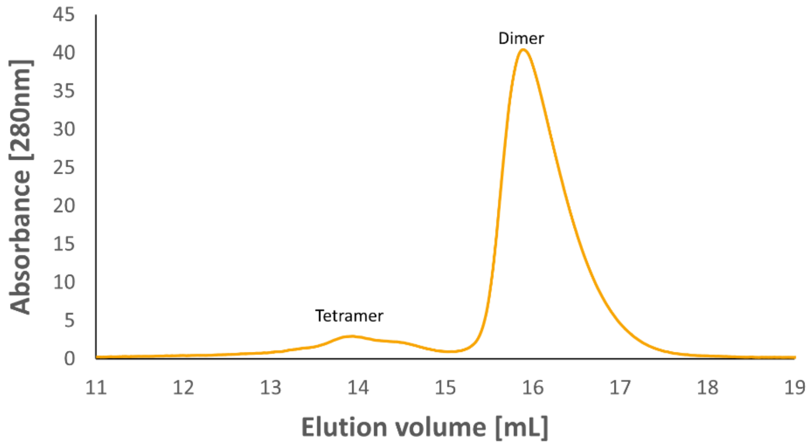

Figure S7. Size exclusion chromatography (SEC) analysis of LpMAT.

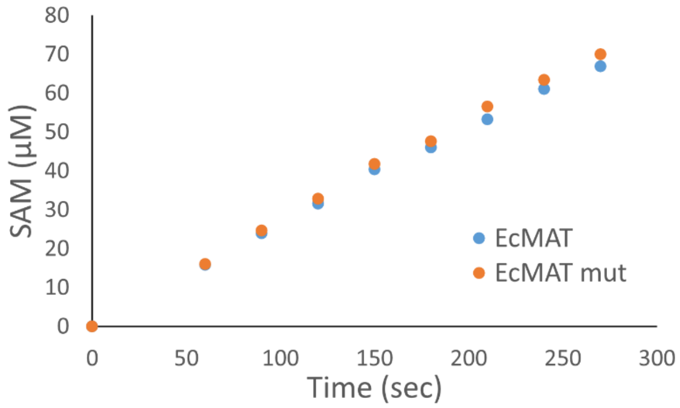

Figure S8. Removal of salt-bridge forming residues in EcMAT does not affect catalytic activity. Time dependent accumulation of SAM by EcMAT (blue) and EcMATmut (orange) activities (see Methods).

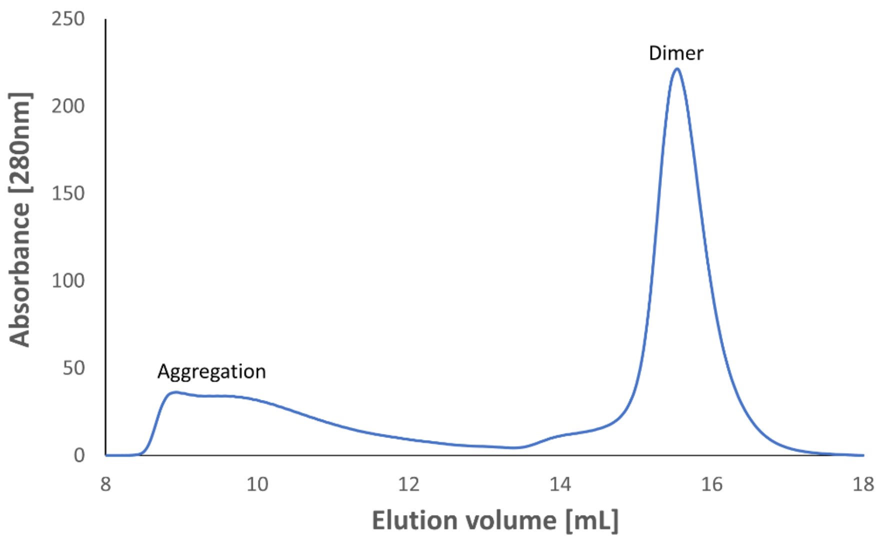

Figure S9. Size exclusion chromatography (SEC) analysis of NgMAT K67E Q98K.

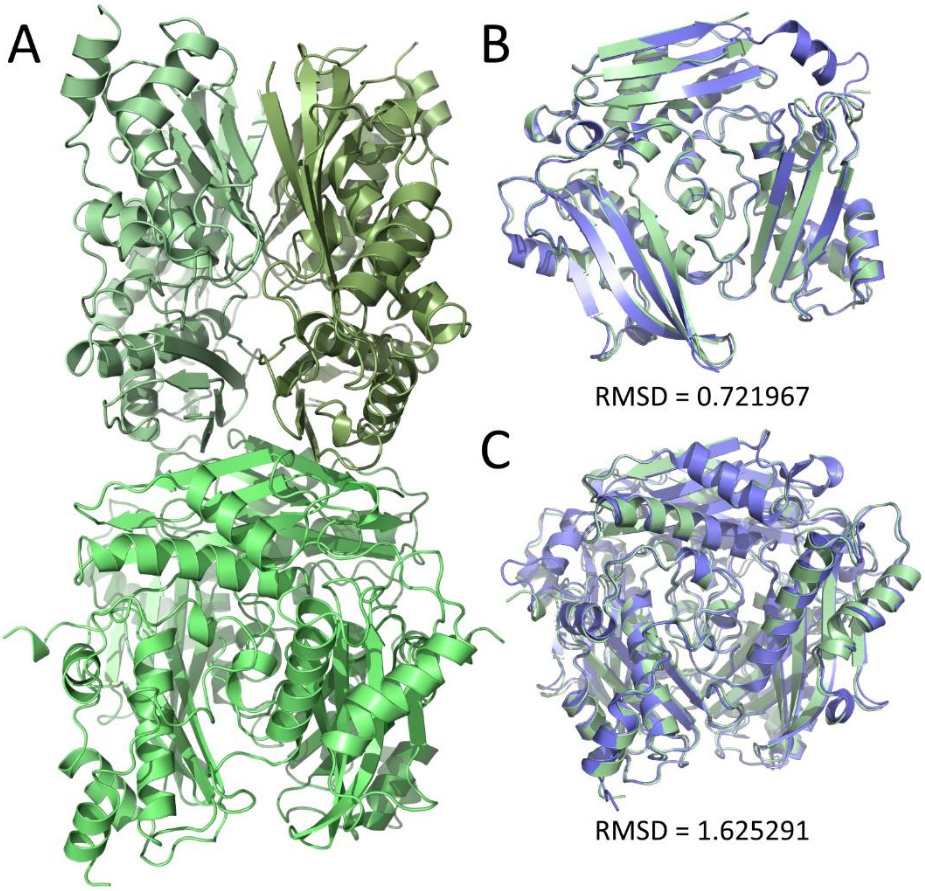

Figure S10. Crystal structure of EcMAT mutant. A. Cartoon representation of EcMATmut homotetramer. Individual subunits are shown in different shades of green. Structural alignments of EcMAT (purple) and EcMATmut (green) monomers **(B)** and dimers **(C)** show very high structural conservation. RMSD, root-mean-square deviation.

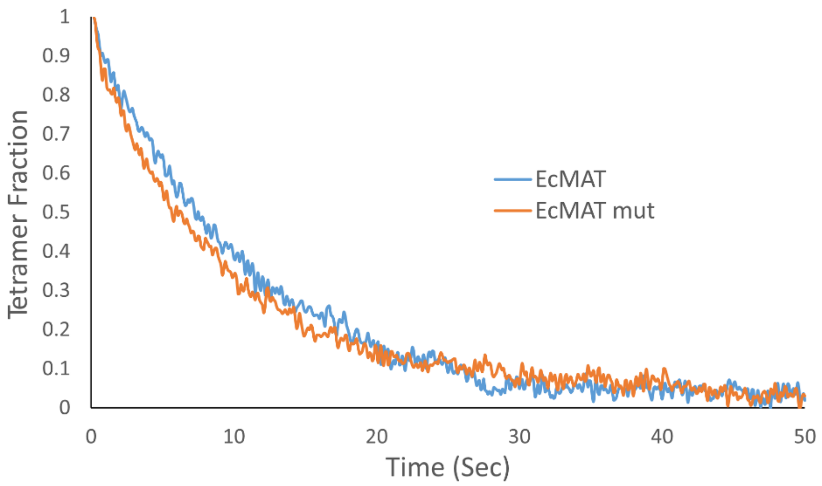

Figure S11. Stopped-flow kinetics of the urea-induced apparent dissociation/unfolding rate of EcMAT (blue) and EcMATmut (orange).

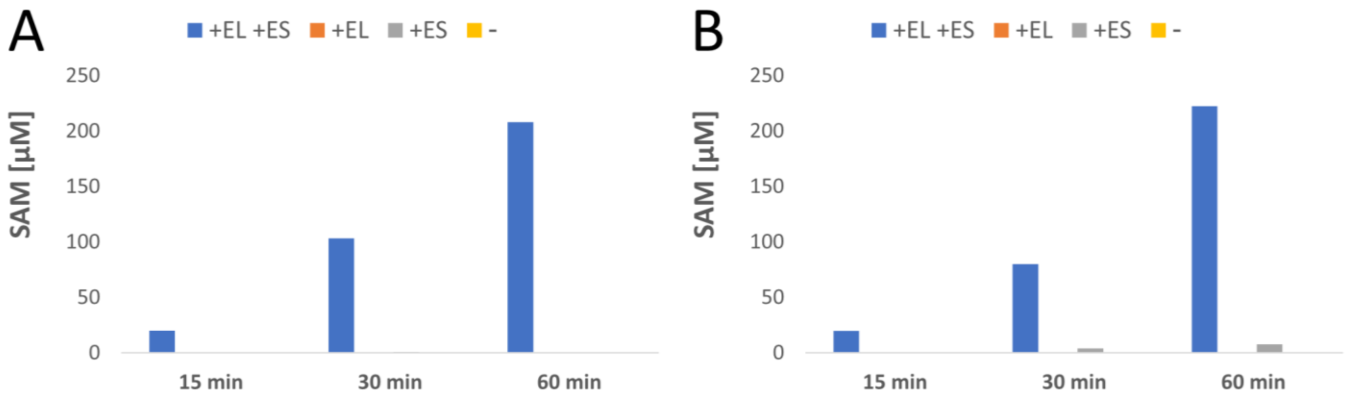

Figure S12. Folding/assembly of EcMAT and EcMATmut is chaperonin-dependent *in vitro*. Accumulation of SAM after diluting GuHCl-unfolded EcMAT **(A)** or EcMATmut **(B)** into refolding/activity mix, containing GroEL/ES system (blue), GroEL only (orange), GroES only (grey) or no chaperones (yellow) (see Methods for details).

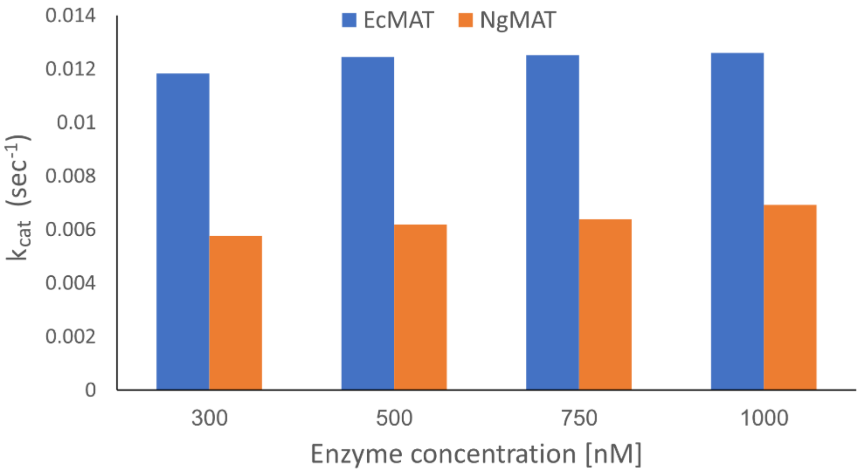

Figure S13. Catalytic turnover of EcMAT and NgMAT is concentration independent. Catalytic activity of EcMAT (blue) and NgMAT (orange) as a function of enzyme concentration.

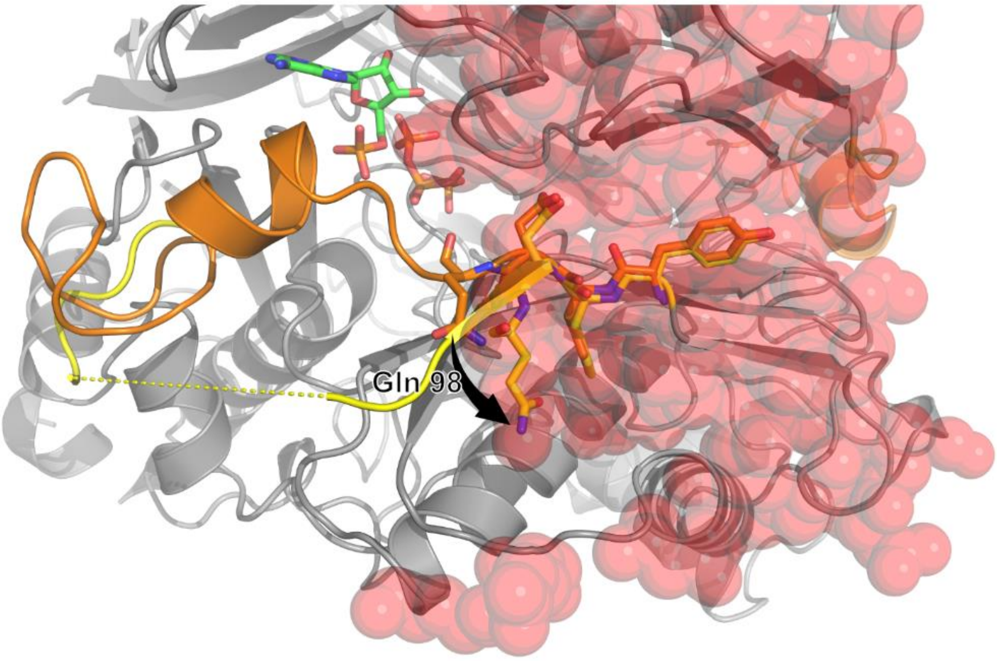

Figure S14. Movement of the active site loop affects the integrity of the inert-dimeric interface in NgMAT. Active site loop in a closed configuration (orange) was superimposed on the active site with an open loop (yellow). Residues 102-113 of the open loop are unresolved. The active site is occupied with AMP and triphosphate (shown in sticks). The movement of the side chain of Gln98 is marked with a black arrow. The small (inter-dimeric) interface is shown in red spheres. The figure was built based on PDB ID 5T8S.
