# Supplementary material for: Evolution of homo‐oligomerization of methionine S‐adenosyltransferases is replete with structure–function constrains
Source: Protein Sci. 2022 Jun 16;31(7):e4352. doi: 10.1002/pro.4352 (PMC9202080; doi:10.1002/pro.4352)
Supplement: Supplementary file 2 — Table S1 List of available bacterial MAT crystal structures [file PRO-31-e4352-s002.docx]

| **Organism** | **PDB** | **Assembly** | **Phylum** | **Class** |
| --- | --- | --- | --- | --- |
| Escherichia coli | 1MXA | Tetramer | Proteobacteria | Gammaproteobacteria |
| Escherichia coli | 1MXB | Tetramer | Proteobacteria | Gammaproteobacteria |
| Escherichia coli | 1MXC | Tetramer | Proteobacteria | Gammaproteobacteria |
| Escherichia coli | 1XRA | Tetramer | Proteobacteria | Gammaproteobacteria |
| Escherichia coli | 1XRB | Tetramer | Proteobacteria | Gammaproteobacteria |
| Escherichia coli | 1XRC | Tetramer | Proteobacteria | Gammaproteobacteria |
| Escherichia coli | 1FUG | Tetramer | Proteobacteria | Gammaproteobacteria |
| Escherichia coli | 1P7L | Tetramer | Proteobacteria | Gammaproteobacteria |
| Escherichia coli | 1RG9 | Tetramer | Proteobacteria | Gammaproteobacteria |
| Escherichia coli | 7LOZ | Tetramer | Proteobacteria | Gammaproteobacteria |
| Escherichia coli | 7LOW | Tetramer | Proteobacteria | Gammaproteobacteria |
| Escherichia coli | 7LOO | Tetramer | Proteobacteria | Gammaproteobacteria |
| Escherichia coli | 7LO2 | Tetramer | Proteobacteria | Gammaproteobacteria |
| Escherichia coli | 7LNN | Tetramer | Proteobacteria | Gammaproteobacteria |
| Escherichia coli | 7LL3 | Tetramer | Proteobacteria | Gammaproteobacteria |
| Escherichia coli K-12  + Escherichia virus T3 | 7OCK | Tetramer | Proteobacteria | Gammaproteobacteria |
| Neisseria gonorrhoeae | 5T8S | Tetramer | Proteobacteria | Betaproteobacteria |
| Neisseria gonorrhoeae | 5T8T | Tetramer | Proteobacteria | Betaproteobacteria |
| Burkholderia pseudomallei | 3IML | Tetramer | Proteobacteria | Betaproteobacteria |
| Mycobacterium tuberculosis | 3TDE | Tetramer | Actinomycetota | Actinomycetia |
| Mycobacterium marinum | 3RV2 | Tetramer | Actinomycetota | Actinomycetia |
| Mycobacterium avium | 3S82 | Tetramer | Actinomycetota | Actinomycetia |
| Thermus thermophilus | 5H9U | Tetramer | Deinococcota | Deinococci |
| Campylobacter jejuni | 4LE5 | Dimer | Campylobacterota | Campylobacteria |
| Ureaplasma urealyticum | 6RKC | Tetramer | Mycoplasmatota | Mollicutes |
| Ureaplasma urealyticum | 6RK7 | Tetramer | Mycoplasmatota | Mollicutes |
| Ureaplasma urealyticum | 6RK5 | Tetramer | Mycoplasmatota | Mollicutes |
| Ureaplasma urealyticum | 6RJS | Tetramer | Mycoplasmatota | Mollicutes |
| Ureaplasma urealyticum | 6RKA | Tetramer | Mycoplasmatota | Mollicutes |

**Table S1. List of available bacterial MAT crystal structures**
