# Supplementary material for: Evolution of homo‐oligomerization of methionine S‐adenosyltransferases is replete with structure–function constrains
Source: Protein Sci. 2022 Jun 16;31(7):e4352. doi: 10.1002/pro.4352 (PMC9202080; doi:10.1002/pro.4352)
Supplement: Supplementary file 3 — Table S2 Crystallographic data collection and refinement statistics [file PRO-31-e4352-s003.docx]

**Table S2. Crystallographic data collection and refinement statistics**

| Structure | EcMetK_E67K_K98Q | MetK_LP |
| --- | --- | --- |
| PDB ID | 7R2W | 7R3B |
| Data collection |  |  |
| Beamline | DLS I03 | DLS I04 |
| Wavelength (Å) | 0.97626 | 0.9795 |
| Resolution range (Å) | 45.49 -1.6 | 29.39-2.82 |
| Space group | P42212 | P1 |
| A, b, c (Å) | 86.61 86.61 90.98 | 58.43, 110.93, 112.66 |
| Α, β, γ (°) | 90 90 90 | 93.82, 104.07, 99.59 |
| R-merge | 0.083 (3.909) | 0.08 (0.429) |
| R-meas | 0.085 (4.069) | 0.113 (0.607) |
| R-pim | 0.017 (1.098) | 0.08 (0.429) |
| CC1/2 | 1.0 (0.340) | 0.996 (0.706) |
| Multiplicity | 45.8 (24.8) | 3.5 (2.4) |
| Completeness (%) | 100 (99.4) | 94.91 (62.72) |
| Mean I/sigma(I) | 27.7 (0.9) | 10.2 (1.6) |
| Refinement |  |  |
| Reflections used in refinement | 46122 (4470) | 61540  (4103) |
| Reflections used for R-free | 2634 (243) | 3105 (231) |
| R-work/R-free | 0.191 / 0.226 | 0.16/ 0.27 |
| Number of non-hydrogen atoms | 3117 | 22699 |
| Macromolecules | 2898 | 22402 |
| Ligands | 34 | 251 |
| Solvent | 185 | 46 |
| Protein residues | 375 | 2938 |
| Rmsd bonds (Å) | 0.017 | 0.020 |
| Rmsd angles (°) | 2.15 | 2.35 |
| Ramachandran favored (%) | 97.04 | 93.18 |
| Ramachandran allowed (%) | 2.96 | 6.71 |
| Ramachandran outliers (%) | 0.00 | 0.1 |
| Average B-factor | 38.92 | 71.29 |
| Macromolecules | 38.61 | 70.67 |
| Ligands | 35.79 | 130.19 |
| Solvent | 44.39 | 51.21 |

Numbers in parentheses indicate statistics for the highest resolution shell
